# Educational escape games in emotion education: effects on learning achievement, emotion regulation strategies, and achievement emotions among upper elementary students

**DOI:** 10.3389/fpsyg.2026.1877695

**Published:** 2026-07-14

**Authors:** Hao-Chiang Koong Lin, Hui-Shan Chang, Yi-Rong Tseng, Tao-Hua Wang, Cheng-Hsuan Li

**Affiliations:** 1 Department of Information and Learning Technology, National University of Tainan, Tainan, Taiwan; 2 Department of Audiology and Speech-Language Pathology, Asia University, Taichung, Taiwan; 3 Graduate Institute of Educational Information and Measurement, National Taichung University of Education, Taichung, Taiwan; 4 National Museum of Natural Science, Taichung, Taiwan

**Keywords:** achievement emotions, educational escape games, emotion regulation, game-based learning, learning achievement, social-emotional learning

## Abstract

**Introduction:**

Upper elementary students face increasing social-emotional challenges during early adolescence, yet traditional lecture-based emotion education may provide limited opportunities for situated emotional practice. Although game-based learning has been widely used to enhance motivation and cognitive outcomes, the effects of educational escape games (EEGs) on emotion regulation strategies and achievement emotions remain insufficiently understood.

**Methods:**

This study used a quasi-experimental mixed-methods design to examine the effects of integrating EEGs into an emotion education curriculum on fifth-grade students' learning achievement, self-reported emotion regulation strategies, and self-reported achievement emotions. Participants were 56 fifth-grade students from two intact classes at an elementary school in southern Taiwan. One class received a 6-week emotion education curriculum integrated with EEGs, whereas the other received teacher-guided questioning and explanation based on the same curriculum content. Quantitative data were collected through pretests and posttests and analyzed using paired-samples t-tests and ANCOVA. Qualitative data, including classroom observations and a focus group interview with seven students from the experimental group, were analyzed using framework analysis to contextualize the quantitative findings.

**Results:**

After controlling for pretest scores, the experimental group demonstrated significantly higher curriculum-based learning achievement than the control group. However, no significant short-term changes were found in students' self-reported cognitive reappraisal or expressive suppression. Regarding achievement emotions, the experimental group reported higher anxiety and lower enjoyment after the intervention. Qualitative findings suggested that students' anxiety was mainly related to time pressure, puzzle difficulty, and uncertainty, and was often accompanied by continued participation, peer discussion, and problem-solving attempts rather than withdrawal.

**Discussion:**

These findings should be interpreted cautiously because a shortened achievement emotions measure was used and the anxiety subscale showed low internal consistency. Overall, EEGs may provide a high-challenge, highly engaging context for emotion education, but game-based learning does not necessarily increase enjoyment.

## 1 Introduction

### 1.1 Background

In contemporary educational environments, students' social-emotional competence is as important as academic achievement. Upper elementary school students are in the early stages of adolescence, a critical developmental period characterized by shifts in peer relationships and increased academic pressure, which pose multiple developmental challenges (Bathelt et al., 2021; Herd et al., 2020; Mastorci et al., 2024). During this stage, students who lack appropriate emotion regulation are more likely to develop internalizing or externalizing behavioral problems, which subsequently affect their school adjustment and academic performance (Green et al., 2021). Therefore, facilitating students' emotion regulation through systematic educational interventions has become a central concern in education.

Social-emotional learning (SEL) has been widely recognized as a theoretical framework for addressing this need, aiming to cultivate core competencies such as self-awareness and self-management, as outlined by the Collaborative for Academic, Social, and Emotional Learning (2020). Emotion regulation is a central dimension of self-management. Extensive empirical research has demonstrated that SEL programs significantly enhance students' social-emotional competence and academic performance (Durlak et al., 2011). However, implementing SEL in educational settings remains challenging. Traditional didactic teaching often falls short of conveying abstract concepts and fails to provide authentic contextualized scenarios that evoke deep emotional engagement and support strategy practices, resulting in reduced student motivation and insufficient internalization of skills (Najjarpour, 2025).

To address these limitations, game-based learning (GBL) has been proposed as an innovative instructional approach that combines contextual authenticity with high engagement levels (Alotaibi, 2024; Plass et al., 2020). It integrates immersive narratives with challenge-based tasks to create highly interactive learning environments. Specifically, GBL can simulate authentic social and psychological dilemmas, allowing students to experience real emotional fluctuations and engage in emotion regulation practices within a “safe-to-fail” environment (Plass et al., 2020). This design not only supports the maintenance of intrinsic motivation under challenging conditions but also provides a feasible pathway for transforming SEL from abstract conceptual instruction into situated practice (Alotaibi, 2024).

Furthermore, achievement emotions play a central role in shaping cognitive engagement and academic performance throughout the learning process (Pekrun, 2006). These emotions, which are directly related to learning activities and outcomes, influence how students allocate cognitive resources and sustain learning efforts (Pekrun, 2006; Pekrun et al., 2002). Therefore, understanding how students manage emotional arousal while maintaining high-quality engagement in challenging GBL contexts is critical for optimizing learning outcomes.

Although GBL has accumulated substantial empirical support for improving learning achievement and motivation (Alotaibi, 2024; Bodnar et al., 2016; Boyle et al., 2016; Gordillo and López-Fernández, 2024; Gyedu et al., 2026; Hainey et al., 2016; Hussein et al., 2019; López-Pernas et al., 2023; Plass et al., 2020; Tokac et al., 2019), its application in emotion education remains underexplored. Existing studies have primarily focused on overall learning outcomes or general social-emotional competence, with limited attention paid to the specific processes of emotion regulation and mechanisms underlying strategy transformation (Gómez-León, 2025). Moreover, previous research has predominantly relied on qualitative analyses or small-sample case studies (Liverman et al., 2025), with relatively few studies employing rigorous quantitative experimental designs to examine the causal effects of game-based interventions (Veldkamp et al., 2020).

This gap highlights the need for mixed-methods research that systematically examines the relationships among learning achievement, emotion regulation strategies, and achievement emotions in GBL contexts, thereby providing stronger empirical support for the design and implementation of SEL.

### 1.2 Theoretical framework and implementation challenges of social-emotional learning

The theoretical foundation of SEL is grounded in educational psychology perspectives that view emotions as cognitive-affective processes involved in learning, motivation, and academic achievement (Pekrun, 2006; Pekrun et al., 2011). From this perspective, emotional competencies are not fixed traits but can be systematically developed through well-designed instructional interventions (Cipriano et al., 2023; Durlak et al., 2011). The Collaborative for Academic, Social, and Emotional Learning framework emphasizes that these competencies can be cultivated through explicit instruction, opportunities for practice, and the integration of SEL into academic curricula, identifying five core domains: self-awareness, self-management, social awareness, relationship skills, and responsible decision-making (Collaborative for Academic, Social, and Emotional Learning, 2020).

Among these, emotion regulation—the processes through which individuals monitor, evaluate, and modify their emotional experiences and expressions (Gross, 2002; John and Gross, 2007)—is a core component of self-management. Through regulatory strategies, individuals can adjust emotional responses at the physiological, cognitive, and behavioral levels and regulate the intensity, frequency, and duration of emotions to align with social norms or personal goals (Boemo et al., 2022; Cole, 2014). Prior research has demonstrated that effective emotion regulation significantly influences academic performance, achievement emotions, and mental health (Ivcevic and Brackett, 2014; John and Gross, 2004; Pekrun et al., 2009).

According to the process model of emotion regulation proposed by Gross and John (2003), individuals regulate their emotional responses through various strategies, among which cognitive reappraisal and expressive suppression are the most widely studied. Cognitive reappraisal is an antecedent-focused strategy that involves reinterpreting emotional situations to alter their impact (Purnamaningsih, 2017), whereas expressive suppression is a response-focused strategy that involves inhibiting outward expression of emotions after they are elicited (Gross and Levenson, 1993). Cognitive reappraisal is associated with better psychological adjustment and academic outcomes (Kobylińska and Kusev, 2019; Kobylińska et al., 2020; Schwerdtfeger et al., 2019; Vally and Ahmed, 2020), whereas expressive suppression is often linked to poorer emotional functioning (Schäfer et al., 2017). Therefore, promoting adaptive strategies, particularly cognitive reappraisal, is considered an important goal of SEL interventions.

From a developmental perspective, fifth-grade students (approximately 10–11 years old) enter early adolescence, a stage characterized by heightened emotional variability and social transitions (Bathelt et al., 2021; Herd et al., 2020). During this period, students are more susceptible to emotional and behavioral difficulties, including school disengagement and increased interpersonal conflict (Green et al., 2021; Laursen et al., 1998). Although SEL programs improve social–emotional competence and academic outcomes (Durlak et al., 2011), their effectiveness remains uneven. Implementation outcomes are highly dependent on curriculum design, instructional practices, and contextual factors (Cipriano et al., 2023). Furthermore, traditional didactic teaching often lacks opportunities for contextualized practice, limiting students' engagement and internalization of emotion regulation strategies (Najjarpour, 2025).

Therefore, developing instructional approaches that provide authentic emotional experiences and opportunities for strategy practice has become a critical priority in advancing SEL.

### 1.3 Achievement emotions

Beyond emotion-regulation strategies, achievement emotions represent a critical affective dimension that influences learning processes. Achievement emotions are emotions directly related to learning activities or outcomes (Pekrun, 2006). According to control-value theory (CVT), these emotions are determined by individuals' perceived control over tasks and the value they assign to those tasks (Pekrun, 2006).

Empirical research indicates that enjoyment, anxiety, and boredom are among the most commonly experienced achievement emotions among elementary and secondary school students in academic contexts (Lichtenfeld et al., 2012). Enjoyment is a positively activating emotion, whereas anxiety and boredom are negatively activating and negatively deactivating emotions, respectively. These emotions influence learning outcomes through different mechanisms: positive emotions facilitate engagement, whereas negative emotions often impair it (Pekrun et al., 2011).

However, the effects of negative emotions are not uniformly detrimental. Within the framework of CVT, negative emotions such as anxiety may exert dual effects depending on the contextual conditions. Although anxiety can increase effort and motivation in certain situations, it may consume cognitive resources and interfere with higher-order processing (Pekrun, 1992; 2006). In contrast, boredom is more consistently associated with disengagement and avoidance behaviors (Pekrun et al., 2010).

Recent research suggests that highly aroused negative emotions may facilitate effort investment under specific conditions. From the perspective of the Biopsychosocial Model of Challenge and Threat, individuals experiencing threat states may maintain task engagement and exert effort when they perceive sufficient coping resources (van Gog et al., 2024). This finding highlights that emotional effects on learning are context-dependent rather than inherently positive or negative, indicating that emotional responses function dynamically in learning environments.

In summary, achievement emotions are not merely learning outcomes but also key mechanisms shaping cognitive engagement and performance (Pekrun, 2006). Understanding how emotional arousal can be regulated and channeled toward productive learning processes remains an important research topic.

### 1.4 Game-based learning as an innovative path for social-emotional learning

To address the challenges of implementing SEL, GBL has emerged as an instructional approach that integrates contextual authenticity with high engagement. Research has indicated that GBL can enhance learning achievement, motivation, and engagement in immersive and interactive environments (Alotaibi, 2024; Bodnar et al., 2016; Boyle et al., 2016; Fotaris and Mastoras, 2019; Gordillo and López-Fernández, 2024; Gyedu et al., 2026; Hainey et al., 2016; Hussein et al., 2019; Köse and Özcan, 2025; López-Pernas et al., 2023; Makri et al., 2021; Martinez et al., 2023; Plass et al., 2020; Sánchez, 2023; Tokac et al., 2019).

Within this framework, escape games represent a specific form of GBL that combines narrative immersion with time-limited problem-solving tasks (Adams et al., 2018; López-Belmonte et al., 2020; Nicholson, 2015; Veldkamp et al., 2020). Grounded in Situated Learning Theory, escape games provide authentic contexts in which learners engage in meaningful tasks that resemble real-world challenges (Lave and Wenger, 1991). Through such designs, escape games pose social and psychological challenges that require learners to regulate their emotions during problem-solving, thereby facilitating the contextualized application of emotion-regulation strategies (Veldkamp et al., 2020).

A key feature of GBL is the “safe-to-fail” environment, which frames failure as part of the learning process (Loderer et al., 2020; Plass et al., 2015). In escape games, time pressure and task difficulty may induce anxiety. However, in a supportive environment, emotional arousal can promote engagement and persistence. This suggests that negative emotions may function as catalysts for strategy use (Aldao et al., 2015), rather than merely as obstacles.

From an educational psychology perspective, the value of escape games lies not only in their motivational appeal but also in their capacity to create emotionally engaging performance contexts (Plass et al., 2020; Veldkamp et al., 2020). Under time pressure and uncertainty, students may experience frustration or anxiety while simultaneously receiving peer support and opportunities for strategy use (Loderer et al., 2020; Pekrun, 2006). In this sense, escape games may provide situated contexts in which emotional arousal becomes part of students' learning behavior and regulation practice (Aldao et al., 2015; Pekrun et al., 2011).

Social interactions also play a crucial role in escape games. Collaborative problem-solving requires communication, negotiation, and perspective-taking, providing opportunities to practice emotion regulation strategies in social contexts (Vygotsky, 1978). Such peer interactions may reduce individual cognitive and emotional loads, supporting both learning and regulation processes (Pekrun, 2006; Pekrun et al., 2011).

In summary, escape games provide an integrated learning environment in which emotional experiences, social interactions, and cognitive engagement are closely intertwined. This environment may allow emotional arousal to become connected to learning engagement and regulation practice rather than functioning solely as a disruptive factor.

### 1.5 Research gap and purpose

Despite the growing empirical support for GBL and educational escape games (EEGs), several important gaps remain. First, at the theoretical level, existing studies have primarily focused on learning outcomes and engagement, with limited examination of emotion regulation processes and achievement emotions in game-based SEL contexts. Second, at the methodological level, prior research has relied predominantly on qualitative or small-scale designs, with relatively few studies employing quasi-experimental or mixed-methods approaches capable of examining intervention effects while controlling for baseline differences (Veldkamp et al., 2020). Third, at the population level, empirical research focusing on upper elementary school students remains limited, despite this group being at a critical developmental stage of emotional and social development (Bathelt et al., 2021).

Accordingly, this study employed a quasi-experimental mixed-methods design to examine the effects of integrating EEGs into an emotion education curriculum on fifth-grade students' learning achievement, emotion regulation strategies, and achievement emotions. Furthermore, this study seeks to clarify how GBL contexts may provide opportunities for students to experience, regulate, and reinterpret task-related emotional arousal, thereby addressing the limitations of traditional didactic emotion education in terms of skill internalization and offering an empirical basis for the practical implementation of SEL.

## 2 Materials and methods

### 2.1 Research design

This study examined the effects of integrating EEGs into an emotion education curriculum on fifth-grade elementary school students' learning achievement, emotion regulation strategies, and achievement emotions.

This study employed a quasi-experimental design, specifically a pretest–posttest nonequivalent control group design. Considering the administrative constraints and pedagogical norms in school settings, intact classrooms were used as the experimental units. Although this design has limitations in terms of random assignment, baseline equivalence was examined using pretest scores, and a one-way analysis of covariance (ANCOVA) was subsequently employed to control for initial group differences, thereby improving the robustness of between-group comparisons (Shadish et al., 2002).

### 2.2 Participants

The participants were 56 fifth-grade elementary school students from a school in southern Taiwan. Using convenience sampling, one classroom was assigned to the experimental group (*n* = 28; 17 males, 11 females) and the other served as the control group (*n* = 28; 16 males, 12 females). The experimental group received a 6-week emotion education curriculum integrated with escape games, whereas the control group received traditional didactic instruction using the same curricular content. To reduce implementation differences, both classes were taught by the same teacher with a professional background in emotional education. Despite the relatively small sample size, single-site intervention studies can provide valuable insights into instructional processes while strengthening internal validity (Cipriano et al., 2023).

The analytical sample varied depending on data completeness across measurements. Learning achievement was analyzed using the full sample (*N* = 56), whereas analytic samples for the emotion regulation questionnaire and achievement emotions questionnaire were defined by valid responses and listwise deletion (see Table 1 for detailed sample sizes and gender distribution). Prior to data collection, all participants and their parents or legal guardians reviewed the study information and provided written informed consent. Student assent was also obtained where appropriate, and students were informed that participation or withdrawal would not affect their grades, academic evaluation, or relationship with the instructor.

**Table 1:** Sample sizes across analyses.

Analysis/ measure	Total *N*	Experimental group, *n* (male/ female)	Control group, *n* (male/ female)
Learning achievement	56	28 (17/11)	28 (16/12)
Emotion regulation questionnaire	49	25 (15/10)	24 (16/8)
Achievement emotions questionnaire	48	25 (15/10)	23 (13/10)

### 2.3 Integrating educational escape games into the emotion education curriculum

Both the experimental and control groups participated in a 6-week emotion education course. The course content, weekly themes, and instructional activity arrangements are presented in Supplementary material 1. The course was conducted once per week, with each session lasting 40 min, and was taught by the same teacher. During the first 15 min of each session, the teacher introduced the weekly theme to both groups through storytelling. This shared instructional phase ensured that students in both groups were exposed to the same core emotion education content before engaging in the subsequent activities.

Over the next 25 min, students in the two groups engaged in different types of learning activities. Students in the control group participated in teacher-guided question-and-answer activities and explanations. Based on the preceding story, the teacher posed questions to guide students' responses and reflections, but students were not required to engage in structured group discussions. The teacher then provided explanations and clarified the key concepts of the weekly lesson. In contrast, students in the experimental group were randomly divided into groups of four to five within the class, with each group using one tablet computer to complete the weekly educational escape room task through the Holiyo interactive platform.

Each weekly game activity consisted of six puzzles related to the emotion education theme of that week. Students were required to collaborate within their groups to discuss, reason, and answer questions, and to complete the task within 20 min to achieve the goal of successfully escaping the room. The weekly puzzle content and item design are presented in Supplementary material 2. In the final 5 min, the teacher conducted a debriefing session, guiding students in the experimental group to review the correct answers to each puzzle, explain the reasoning behind the solutions, and connect the puzzle content to the emotion education concepts covered that week.

The EEGs were developed using the Holiyo interactive platform. Their design adhered to the SAFE framework (sequenced, active, focused, explicit) (Durlak et al., 2011), ensuring that instructional content was logically structured, actively practiced, thematically focused, and explicitly reinforced through reflection.

Specifically, the weekly puzzles were arranged in a sequence from simple to difficult, enabling students to understand the course content in a progressive manner. The puzzle-solving process required students to actively engage in group discussion, judgment, and decision-making. Each weekly puzzle focused on the emotion education theme of that week. Finally, through post-activity summarization and discussion, the teacher explicitly reinforced students' understanding of the weekly emotion education content. Therefore, the EEGs in this study were not merely used as activities to enhance enjoyment, but rather as structured learning tasks designed to promote students' understanding, application, and reflection on emotion education content.

When designing the six EEG puzzles for each week, as shown in Supplementary material 2, the teacher followed a fixed hierarchy of difficulty. The first two puzzles were basic recall items, mainly used to examine whether students could remember key terms, story content, or basic concepts taught in the lesson, such as the concepts of the “upstairs brain” and “downstairs brain.” The third and fourth puzzles were conceptual understanding items, mainly used to examine whether students could understand the core concepts of the weekly lesson, such as emotional intensity, empathy, and healthy stress. The fifth puzzle was a situational application item, in which students were required to judge more appropriate emotional responses or regulation strategies based on everyday life situations. The sixth puzzle was an integrative reasoning item, in which students needed to summarize key points from the story, activities, or course content and integrate multiple concepts for reasoning. Thus, this item represented the most difficult type of puzzle in each weekly set.

In the EEGs, students solved the puzzles in a sequence from simple to difficult; that is, the order of puzzle solving progressed from basic recall items to conceptual understanding items, situational application items, and finally integrative reasoning items. This progressive design was intended to prevent students from experiencing an excessive problem-solving burden at the beginning of the game and to help them gradually move from recalling course content to understanding concepts, applying them to situations, and ultimately engaging in integration and reasoning. When students encountered difficulties during the puzzle-solving process, they could use the hint function on the Holiyo platform to obtain clues. These hints did not directly provide the answers. Instead, they guided students to recall the course content, reread the clues in the questions, or focus on key concepts, thereby supporting continued group discussion and problem-solving.

#### 2.3.1 Game design and implementation

**Figure 1:**
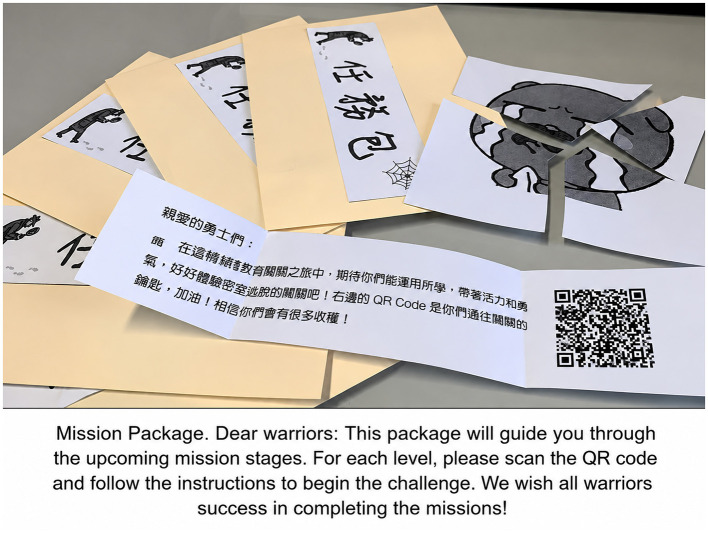
Weekly mission package with QR code.

After the teacher instructed students in the experimental group on how to use the Holiyo platform, the teacher distributed the weekly mission package to each group. Students were asked to read the mission instructions first and then use a tablet computer to scan the QR code (Figure 1) to access the weekly mission and enter the game. The game incorporated narrative elements by positioning students as collaborative problem-solving partners who worked together to complete task-based challenges. In other words, students were required to complete the game tasks through group discussion, collaborative reasoning, and problem-solving. Before the game began, the teacher introduced the narrative scenario to create a challenging atmosphere and encourage students to engage in collaborative problem solving. This design aimed to ensure that students understood the mission goals and rules for collaboration before entering the game.

**Figure 2:**
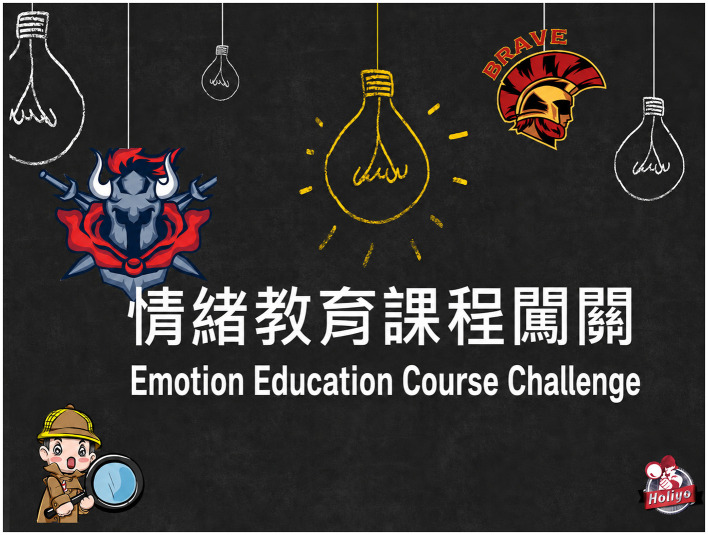
Game cover page.

**Figure 3:**
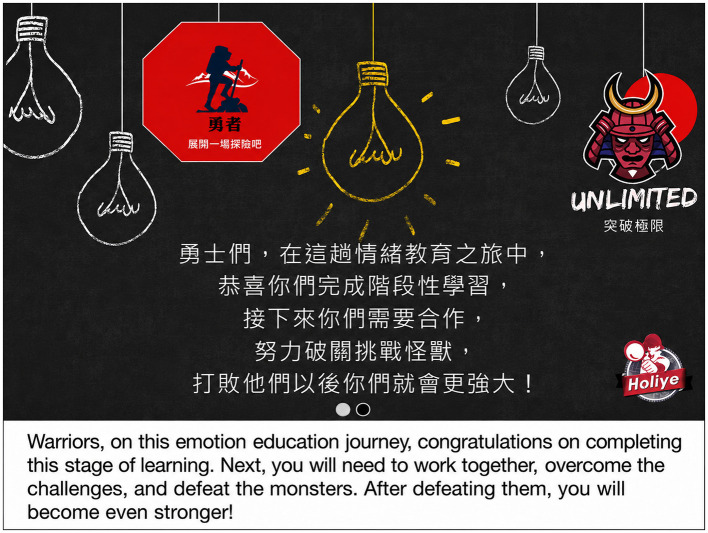
Initial game instruction page.

**Figure 4:**
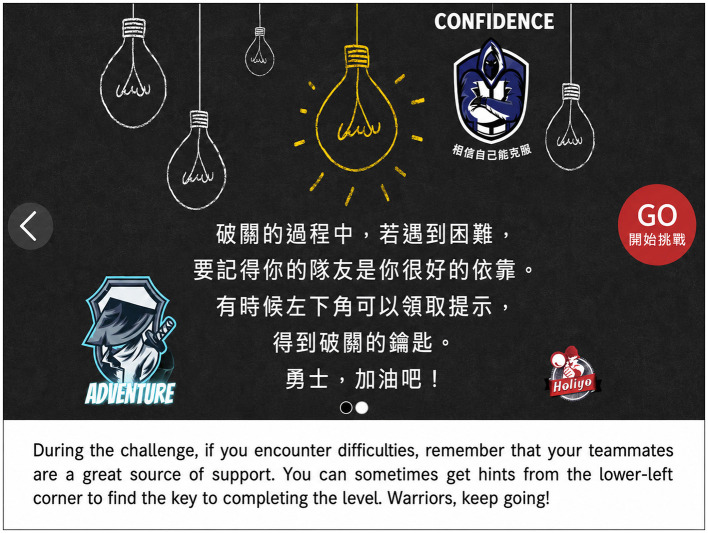
Hint-use and challenge instruction page.

After entering the game, students first viewed the game cover page (Figure 2), followed by the instruction pages (Figures 3, 4), which helped them understand the weekly mission background, challenge goals, and operating procedures. In the game scenario, students were positioned as “warriors” who needed to collect equipment through weekly challenge tasks and gradually prepare for the final challenge. After completing the six weekly missions, the collected equipment symbolized that students had acquired the ability to face the final monster challenge. Within this narrative framework, the weekly puzzles served as the tasks that students needed to complete in order to obtain the equipment. Before the game began, the teacher also reminded students that all puzzles were related to the content of the weekly emotion education lesson and had to be completed through group collaboration, discussion, and reasoning.

**Figure 5:**
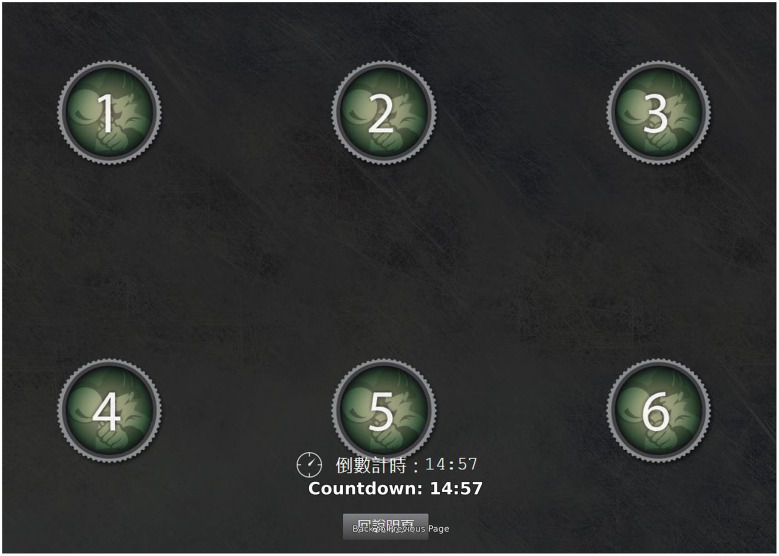
Countdown timer.

**Figure 6:**
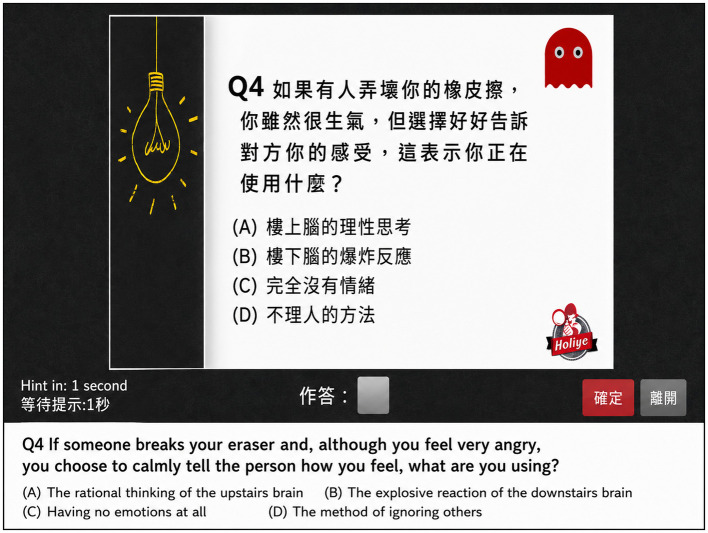
Puzzle page and hint option.

When students entered the puzzle menu, the system activated a countdown timer (Figure 5), creating a time limit for the mission and generating a sense of urgency and challenge characteristic of escape room activities. During the puzzle-solving process, the system presented the puzzle pages and hint options in sequence (Figure 6). To maintain collaborative learning, the teacher required each group member to propose his or her own ideas or judgments for each puzzle before the group discussed and decided on the final answer together. This rule was intended to prevent a small number of students from dominating the problem-solving process and to promote each member's participation in discussion, expression of opinions, and reasoning.

**Figure 7:**
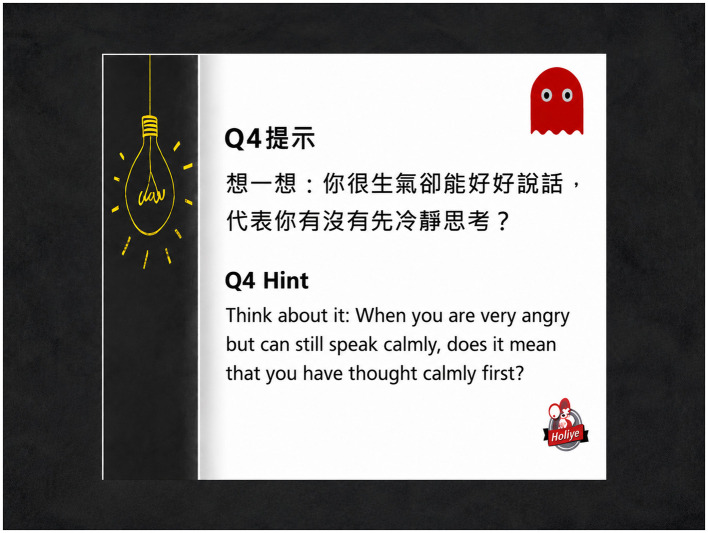
Hint page.

When students encountered a difficult puzzle, the teacher required them to first try to think through the problem and discuss it with their group members. Only when the group had attempted discussion for more than 1 min but was still unable to answer were students allowed to click the hint option in the lower-left corner of the interface to obtain clues (Figure 7), which helped them continue the problem-solving process. These hints did not directly provide the answers. Instead, they guided students to reread the clues in the questions, recall the course content, or think about concepts related to the weekly emotion education theme.

**Figure 8:**
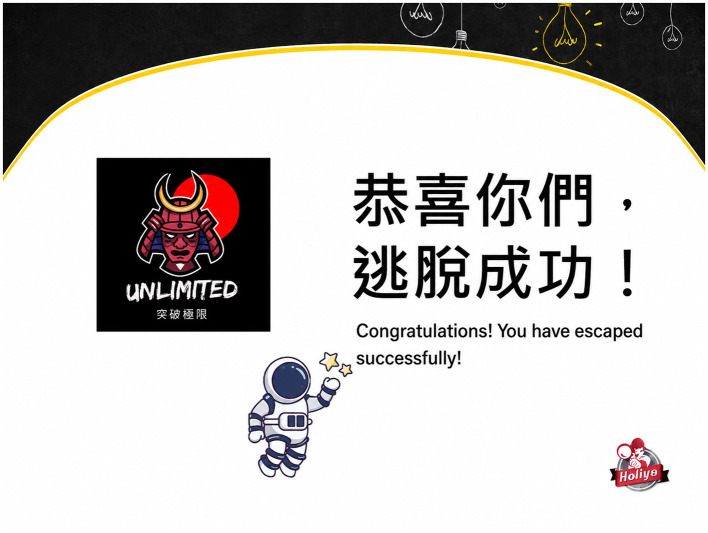
Mission success page.

After students answered at least five of the six puzzles correctly, the system displayed the successful escape screen (Figure 8). If a group answered fewer than five puzzles correctly, the challenge was considered unsuccessful. After successfully escaping, students received one piece of symbolic equipment. Over the 6-week course, students gradually collected equipment by completing the weekly missions. If they completed all six weekly missions, they obtained a complete set of equipment, representing the narrative outcome of being prepared to face the final monster challenge. This reward mechanism extended the game narrative, allowing students to gradually accumulate mission achievements throughout the 6-week course and enhancing their motivation to participate in subsequent course activities.

During the challenge activities, the teacher also walked around the classroom to ensure that students were engaging in group collaboration to solve the puzzles. The teacher mainly observed each group's discussion process and collaborative interactions and confirmed that every member participated in group problem-solving. The teacher did not directly provide answers. Instead, when necessary, the teacher reminded students to return to the hints in the questions, the course content, or the group discussion rules, thereby maintaining the challenge of the game activity and the characteristics of collaborative learning.

### 2.4 Research procedure

**Figure 9:**
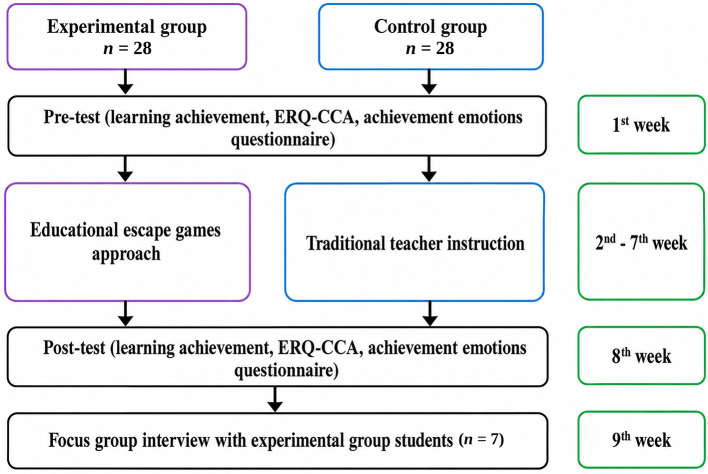
Research flowchart.

This study was conducted in four stages (Figure 9). In the first stage baseline data were collected through pretests, including assessments of learning achievement, emotion regulation strategies, and achievement emotions. The second stage involved a 6-week instructional intervention. The experimental group participated in EEG-based activities, whereas the control group received traditional instruction, with both groups following the same course content (Supplementary material 1). In the third stage, posttests were administered to evaluate differences in learning outcomes, emotion regulation strategies, and achievement emotions. Finally, semi-structured focus group interviews were conducted with seven students from the experimental group. The interviews were audio-recorded, transcribed verbatim, and analyzed to understand students' learning experiences, strategy use, emotional responses, and possible application of course content in relation to the EEG activities.

### 2.5 Research instruments

Both quantitative and qualitative instruments were used to evaluate instructional effects and explore students' psychological experiences during the game-based emotion education activities.

#### 2.5.1 Quantitative instruments

##### 2.5.1.1 Learning achievement questionnaire

This questionnaire was developed by the authors based on instructional objectives and consisted of 20 multiple-choice items provided in Supplementary material 3. To ensure measurement quality, two experienced fifth-grade teachers reviewed the content validity and evaluated item alignment with students' language comprehension and cognitive understanding of escape games and emotion education; minor wording revisions were made to enhance face validity and representativeness.

##### 2.5.1.2 Emotion regulation questionnaire

The Chinese adaptation of the Emotion Regulation Questionnaire for Children and Adolescents (ERQ-CCA) (Liu et al., 2017) was used to assess emotion regulation strategies and is provided in Supplementary material 2. This questionnaire was adapted from the Emotion Regulation Questionnaire for Children and Adolescents (ERQ-CA) (Gullone and Taffe, 2012) for individuals aged 7 to 18 years. Its theoretical foundation can be traced back to the adult Emotion Regulation Questionnaire (ERQ) (Gross and John, 2003) based on the process model of emotion regulation. To reduce the cognitive load for younger respondents, ERQ-CCA simplified the item wording and adopted a 5-point Likert scale (1 = strongly disagree, 5 = strongly agree). Liu et al. (2017) demonstrated that this Chinese version possesses strong psychometric properties and includes 10 items across two dimensions: cognitive reappraisal (six items) and expressive suppression (four items), with Cronbach's α of 0.754 and 0.635, respectively.

##### 2.5.1.3 Achievement emotions questionnaire

Based on CVT, this study selected and adapted items from the elementary school version of the Achievement Emotions Questionnaire (AEQ-ES) (Lichtenfeld et al., 2012). The item content is provided in Supplementary material 3. To reduce cognitive load and assessment fatigue, five items were ultimately included in this study: two items measuring enjoyment and three items measuring anxiety. Responses were rated on a 5-point Likert scale ranging from 1 = strongly disagree to 5 = strongly agree. Two experienced teachers reviewed the items for content validity to ensure that they were appropriate for fifth-grade students' contexts and cognitive levels. The original AEQ-ES demonstrated reliability coefficients ranging from 0.70 to 0.95 (Lichtenfeld et al., 2012). Given the limitations of the shortened scale, this study focused on two core dimensions: enjoyment and anxiety. Boredom was originally measured using only a single item; therefore, it was not included in the subsequent analyses or interpretation of the results.

Because this study involved repeated measurements in an elementary school classroom context, students' age, response time, and cognitive load were considered. Accordingly, simplified items were used to reduce the response burden. However, this approach also limited the psychometric strength of the achievement emotion measures. In particular, the internal consistency of the anxiety subscale was relatively low, and boredom was measured with only a single item. Therefore, the achievement emotion results in this study were regarded as preliminary and exploratory self-reported emotional responses and were interpreted with caution.

#### 2.5.2 Qualitative instrument

##### 2.5.2.1 Observation records

During the implementation of the six course sessions, the classroom teacher and the school counseling teacher conducted naturalistic classroom observations of students in both the experimental and control groups to record their observable behaviors in different instructional contexts. The classroom observations focused on students' interactions with the teacher and peers, level of classroom participation, and observable emotional responses. After each session, the classroom teacher wrote observation notes and instructional summaries, which served as references for describing classroom implementation, understanding students' learning responses, and improving subsequent instructional activities.

##### 2.5.2.2 Semi-structured interview guide

To further contextualize the quantitative findings, a focus group interview was conducted with seven students from the experimental group, lasting approximately 45 min. The semi-structured interview guide (Supplementary material 4) was reviewed by experts and covered four main areas of inquiry to elicit students' responses and reflections: perceived improvements in learning and changes in achievement, the use or shift of emotion regulation strategies, experiences of stress and emotion management, and expectations before learning and reflections after learning.

This study adopted framework analysis (Gale et al., 2013; Ritchie et al., 2003). Based on the research objectives of this study, the analytical framework was systematically predefined with four core framework categories: learning improvements, shift strategies, stress coping, and learning perceptions (Table 2).

**Table 2:** Axial coding categories and descriptions derived from student interviews.

Code	Axial coding category	Descriptions	Open codes
C1	Learning improvements	Improvements of learners from the emotion regulation course	Skill acquisition; learning achievement; application
C2	Shift strategies	Impact on learners' emotion regulation strategy use	Strategy application; cognitive shift
C3	Stress coping	Impact on learners' stress management	Underlying emotion; emotion suppression
C4	Learning perceptions	Learners' perceptions of the course	Pre-learning expectations; post-learning reflections

### 2.6 Data analysis

This study adopts a concurrent mixed-methods analytical strategy to ensure the rigor of the research conclusions. The specific analytical procedure is described below.

#### 2.6.1 Quantitative analysis

All quantitative analyses were conducted using IBM SPSS Statistics version 25.0 (IBM Corp., Armonk, NY, United States). Descriptive statistics, including means and standard deviations, were calculated for all study variables. The internal consistency of the ERQ-CCA and the AEQ-ES subscales included in the main analyses was assessed using Cronbach's α. For the AEQ-ES, reliability analyses were conducted only for the enjoyment and anxiety subscales. Within-group pretest–posttest changes were analyzed using paired-samples *t*-tests, while between-group intervention effects were tested using one-way analysis of covariance (ANCOVA), with pretest scores entered as covariates. Before conducting the ANCOVA, the assumption of homogeneity of regression slopes was tested. After confirming that this assumption was not violated, the main analyses were performed. Effect sizes were interpreted according to the criteria proposed by Cohen (1988), including Cohen's *d* and partial eta squared ($\eta_{\text{p}}^{2}$).

#### 2.6.2 Qualitative analysis

As described in Section 2.5.2.2, this study employed framework analysis (Gale et al., 2013; Ritchie et al., 2003) to systematically analyze 88 valid responses generated from the audio recordings and verbatim transcripts. The analysis was conducted in two stages. The first stage involved initial coding, during which the author and the school counseling teacher independently conducted a conceptual review of the response texts to identify conceptual units and preliminary themes. The second stage involved framework coding, in which the conceptual units were systematically mapped onto four predefined framework categories: learning improvements, shift strategies, stress coping, and learning perceptions (Table 2).

To enhance the trustworthiness of the qualitative findings, this study adopted procedures of independent coding, peer checking, and consensus discussion (Lincoln and Guba, 1985; Miles and Huberman, 1994). The two coders first independently evaluated the 88 valid statements and classified them according to the coding results from the previous stage and the four predefined framework categories described above. The initial independent coding results showed that the two coders agreed on the classification of 79 statements, yielding an initial percentage agreement of 89.77%. For the remaining nine statements that involved minor conceptual discrepancies, the author and the school counseling teacher held a consensus meeting to re-examine the contextual meanings of the students' statements and their category assignments. These statements were included in the final analysis after consensus was reached.

Methodological triangulation was applied by integrating quantitative results with qualitative findings from interviews and observations to explain underlying psychological processes and enhance the validity and interpretive depth of the mixed-methods design.

## 3 Results

This study investigated the effects of integrating EEGs into emotion education curricula on fifth-grade elementary school students' learning achievement, emotion regulation strategies, and achievement emotions. The analysis followed a multi-stage approach: (a) descriptive statistics and reliability verification; (b) within-group and between-group comparisons using paired-samples *t*-tests and one-way ANCOVAs; and (c) qualitative analyses used to contextualize and interpret the quantitative findings.

### 3.1 Quantitative results

#### 3.1.1 Descriptive statistics

A total of 56 students participated in this study (experimental group, *n* = 28; control group, *n* = 28). Learning achievement was analyzed using the full sample (*N* = 56).

A total of 49 valid responses were obtained for the emotion regulation questionnaire (experimental group, *n* = 25; control group, *n* = 24). Cronbach's α for cognitive reappraisal was 0.754, meeting the acceptable criterion recommended by DeVellis and Thorpe (2021) (α ≥ 0.70), indicating reliable internal consistency. Cronbach's α for expressive suppression was 0.635, falling within the questionable range (0.60 ≤ α <0.70) (DeVellis and Thorpe, 2021). Given the exploratory design of this study and the limited sample size (*n* = 49), the results for this subscale should be interpreted with caution and regarded as preliminary evidence requiring validation in future research.

The sample size for the achievement emotions questionnaire was 48 students (experimental group, *n* = 25; control group, *n* = 23). Cronbach's α for the enjoyment subscale was 0.698, which was close to the acceptable level of 0.70. The anxiety subscale consisted of three items, with a Cronbach's α of 0.571, indicating relatively low internal consistency. Considering that Cronbach's α may be affected by the small number of items, this study further examined the mean inter-item correlation (MIC) of the anxiety subscale. The results showed that the MIC values for the anxiety subscale ranged from 0.346 to 0.419, falling within the range of item associations often considered acceptable in studies using short scales (Briggs and Cheek, 1986; Clark and Watson, 1995). Therefore, although the Cronbach's α of the anxiety subscale did not reach the ideal level, the items still showed reasonable inter-item associations. Given this limitation, the anxiety-related findings in this study were regarded as preliminary and exploratory self-reported emotional responses and were interpreted with caution. These findings were primarily contextualized through classroom observations and focus group interviews rather than used as the sole basis for strong causal or mechanistic conclusions.

#### 3.1.2 The effects on learning achievement

Table 3 summarizes the learning-achievement results. Paired-samples *t*-test showed that the experimental group's mean scores improved significantly from 62.68 to 77.86, *t*_(27)_ = −6.14, *p* < 0.001, *d* = 1.16, representing a large effect size (Cohen, 1988). The control group also improved significantly from 62.32 to 68.93, *t*_(27)_ = −2.74, *p* < 0.05, *d* = 0.52, indicating a medium effect size (Cohen, 1988).

**Table 3:** Descriptive statistics for learning achievement.

Variables	Group	Pretest *M* (*SD*)	Posttest *M* (*SD*)	*t* values	*F* values
Learning achievement	Experimental	62.68 (14.24)	77.86 (18.08)	−6.14^**^	6.32^*^
	Control	62.32 (13.09)	68.93 (16.01)	−2.74^*^	

Single asterisk indicates p <0.05; double asterisks indicate p <0.001.

One-way ANCOVA was conducted after confirming the homogeneity of the regression slopes, *F*_(1, 52)_ = 0.19, *p* = 0.668. Results indicated a significant main effect of group, *F*_(1, 53)_ = 6.32, *p* < 0.05, $\eta_{\text{p}}^{2}$ = 0.107, with a large effect size (Cohen, 1988). This indicates that after controlling for pretest scores, students in the escape games condition demonstrated significantly higher learning achievement than those in the traditional instruction condition.

#### 3.1.3 The effects on emotion regulation strategies

Table 4 presents the statistics for emotion regulation strategies. In the experimental group, neither cognitive reappraisal [*t*_(24)_ = 0.38, *p* = 0.707], nor expressive suppression [*t*_(24)_ = 0.88, *p* = 0.387], showed any significant changes. Similarly, the control group exhibited no significant shift in either dimension (*p*s > 0.05).

**Table 4:** Descriptive statistics for emotion regulation.

Variables	Group	Pretest	Posttest	*t* values	*F* values
		*M* (SD)	*M* (SD)		
Cognitive reappraisal	Experimental	4.01 (0.57)	3.95 (0.54)	0.38	0.97
	Control	4.20 (0.67)	4.11 (0.76)	0.49	
Expressive suppression	Experimental	3.02 (0.95)	2.83 (0.81)	0.88	1.97
	Control	2.64 (0.80)	2.49 (0.85)	0.54	

After confirming the homogeneity of the regression slopes for both dimensions (*p*s > 0.05), ANCOVA results revealed no significant between-group differences in either cognitive reappraisal, *F*_(1, 46)_ = 0.97, *p* = 0.330, or expressive suppression, *F*_(1, 46)_ = 1.97, *p* = 0.168. These results indicate that this study did not observe significant short-term differences between the two instructional approaches in students' self-reported emotion regulation strategies. However, because the internal consistency of the expressive suppression subscale was relatively low (Cronbach's α = 0.635), the nonsignificant findings related to expressive suppression should be interpreted with caution and should not be taken as evidence that different instructional interventions had no effect on this dimension.

#### 3.1.4 The effects on achievement emotions

As shown in Table 5, the experimental group's enjoyment decreased significantly from 4.38 to 3.92, *t*_(24)_ = 2.43, *p* < 0.05, *d* = −0.49, while anxiety increased significantly from 1.79 to 2.97, *t*_(24)_ = −6.32, *p* < 0.001, *d* = 1.26. In the control group, enjoyment remained stable, *t*_(22)_ = −0.75, *p* = 0.460, but anxiety increased significantly from 2.07 to 2.51, *t*_(22)_ = −2.15, *p* < 0.05, *d* = 0.45.

**Table 5:** Descriptive statistics for achievement emotions.

Variables	Group	Pretest *M* (SD)	Posttest *M* (SD)	*t* values	*F* values
Enjoyment	Experimental	4.38 (0.58)	3.92 (0.80)	2.43^*^	5.76^*^
	Control	4.26 (0.92)	4.41 (0.69)	−0.75	
Anxiety	Experimental	1.79 (0.62)	2.97 (0.57)	−6.32^**^	5.32^*^
	Control	2.07 (0.75)	2.51 (0.82)	−2.15^*^	

Single asterisk indicates p <0.05; double asterisks indicate p <0.001.

ANCOVA results revealed significant between-group differences for enjoyment, *F*_(1, 45)_ = 5.76, *p* < 0.05, $\eta_{\text{p}}^{2}$ =0.114, and anxiety, *F*_(1, 45)_ = 5.32, *p* < 0.05, $\eta_{\text{p}}^{2}$ = 0.106, both with medium effect sizes. Specifically, the control group reported higher adjusted enjoyment, whereas the experimental group reported higher adjusted anxiety, indicating different emotional responses to the two instructional conditions.

### 3.2 Qualitative results

#### 3.2.1 Qualitative summary of classroom observations

The classroom observation results identified three main findings related to the intervention context. First, regarding classroom interaction, students in the experimental group more frequently engaged in discussions and interactions with the teacher and their peers and were able to actively respond to the teacher's questions. Some students demonstrated cognitive engagement and a tendency toward perspective-taking when answering questions, and they actively sought assistance from the teacher or peers when encountering difficulties.

Second, regarding classroom participation, students in the experimental group showed a high level of concentration during the EEG tasks and quickly adapted to the digital puzzle-solving environment. During the group tasks, students collaboratively read the questions, discussed hints, exchanged ideas, and attempted to form shared answers. In contrast, students in the control group mainly participated in teacher-guided question-and-answer and explanation activities, and their classroom participation was less consistent. Some students remained attentive and responded to the teacher's questions, whereas others were more easily distracted or less likely to participate actively.

Third, regarding observable emotional responses, students in the experimental group displayed noticeable tension and a sense of urgency during the puzzle-solving process, particularly when facing the countdown timer, more difficult questions, or concerns that their group might not successfully complete the challenge. However, these emotional responses were generally accompanied by continued task engagement and group collaboration, such as discussing answers, checking hints, or reminding group members to manage time, rather than by withdrawal or giving up. In comparison, the classroom atmosphere in the control group was generally more stable, and students' observable emotional responses were less pronounced. Therefore, the teacher mainly judged students' classroom participation by observing whether they responded to questions, listened attentively, and completed classroom tasks.

#### 3.2.2 Interview content analysis

This study systematically categorized the interview responses of seven students from the experimental group into four framework categories (Table 2) to provide an in-depth contextualization and interpretation of their learning experiences, emotional responses, strategy use, and learning perceptions during the EEG activities.

##### 3.2.2.1 C1: learning improvements

Within the category of learning improvements, students in the experimental group mainly described their understanding of emotion recognition, emotion regulation methods, and the applicability of the course content after the intervention. Some students mentioned that completing the game tasks and successfully solving the puzzles gave them a sense of achievement and increased their attention to the course content. E1, E5, and E6 indicated that they regarded “counting to 10” as an emotion regulation method that could help them calm down. E4 stated that he or she enjoyed looking for facial cues and considered this helpful for recognizing emotions in oneself and others. In addition, E6 mentioned that he or she liked the concepts of the “upstairs brain” and “downstairs brain” because this classification provided a simple and clear way to understand the relationship between different brain functions and emotional responses. These responses suggest that students were able to explain the specific uses of what they had learned in the course and connect some course concepts to everyday emotional situations.

##### 3.2.2.2 C2: shift strategies

Within the category of shift strategies, most students in the experimental group described attempting to use different methods to regulate their emotional states when facing stress or negative emotions. E4 mentioned that he or she would go for a walk or leave the situation to regulate emotions. E1 stated that he or she used the physical action of “wringing a cloth” as a way to express or release emotions such as anger or unhappiness. Regarding cognitive shifting or cognitive restructuring, E2 and E5 indicated that when they felt uncomfortable or emotionally low, they would try to use certain methods to shift their mood and reduce internal discomfort. E7 mentioned that he or she would actively discuss problems with family members or friends, reflecting a tendency to seek support and solve problems through communication. Overall, these responses suggest that most students in the experimental group were able to notice their own emotional states and describe possible emotion regulation methods, including temporarily leaving the situation, physical release, cognitive shifting, and discussing problems with others.

##### 3.2.2.3 C3: stress coping

Within the category of stress coping, most students in the experimental group described their emotional experiences and coping methods when facing stress or difficult situations. E3 reported feeling uneasy when encountering stress in daily life but stated that he or she would try to overcome it. E4 also mentioned that stressful situations might evoke feelings of anxiety or uneasiness. These responses indicate that some students were able to notice and describe their internal emotions and would attempt to respond to or regulate stress in different ways. For example, E1 mentioned that he or she would express emotions through facial expressions and shift his or her mood by watching television or playing with a younger brother. E4 stated that when feeling unhappy, he or she might express emotions through bodily movements and would first play online games to regulate stress. E6 mentioned that he or she would first count from 1 to 10 to calm down and then play with a younger sister. E7 stated that he or she would talk to family members about feelings and seek emotional support. Overall, these self-reported responses suggest that most students in the experimental group were able to identify feelings of stress and describe how they might express emotions through facial expressions, bodily movements, or verbal communication, rather than completely suppressing their feelings. In addition, they mentioned several possible coping methods, including attentional distraction, pausing before reacting, regulation through games or activities, and seeking support from others.

##### 3.2.2.4 C4: learning perceptions

Within the category of learning perceptions, most students in the experimental group reported that, before the course began, they looked forward to the integration of EEGs with the emotion education course. E4 and E6 also mentioned that they felt happy before the course, suggesting that some students held positive expectations toward this course format. After the course, most students indicated that learning content related to emotion regulation helped them understand or regulate their emotions. For example, E2 stated that after completing the course, he or she found it interesting to realize that there were many ways to help regulate emotions and that the course helped him or her express unhappy feelings rather than remaining unhappy for a long time. E3 and E4 indicated that the course helped them become more aware of emotional fluctuations and understand ways to release stress or regulate emotions. E5 stated that he or she was happy to learn new knowledge. E6 indicated that, after learning the course content, these strategies could help him or her regulate unhappy emotions. E7 stated that, after the course, he or she tried to express personal thoughts and believed that this might help reduce conflicts and misunderstandings with family members. Overall, these responses suggest that most interviewed students held positive attitudes toward the course and perceived the course content as helpful for understanding, expressing, and regulating emotions.

### 3.3 Summary

In summary, this study integrated quantitative statistical data and qualitative practical findings through triangulation and identified four main findings. First, regarding learning achievement, students in both groups showed improvement on the posttest. However, after controlling for pretest scores, the experimental group demonstrated significantly higher learning achievement than the control group, suggesting that integrating EEGs into the emotion education course may have contributed to significantly greater learning gains.

Second, regarding emotion regulation strategies, neither group showed significant within-group or between-group differences in self-reported cognitive reappraisal or expressive suppression scores. This result indicates that this study did not observe significant short-term effects of the two instructional approaches on students' self-reported emotion regulation strategies. However, because the internal consistency of the expressive suppression subscale was relatively low, the nonsignificant findings for this dimension should be interpreted with caution.

Third, regarding achievement emotions, students in the experimental group showed a significant decrease in enjoyment and a significant increase in anxiety. Anxiety also increased in the control group. However, between-group comparisons showed that the experimental group reported higher adjusted posttest anxiety, whereas the control group reported higher adjusted posttest enjoyment. Because the Cronbach's α of the anxiety subscale did not reach an ideal level, these findings should be regarded as preliminary and exploratory self-reported emotional responses. Classroom observations further indicated that students' tension and sense of urgency during the puzzle-solving process in the experimental group were generally accompanied by continued task engagement, group discussion, and collaboration, rather than withdrawal or giving up.

Fourth, the qualitative interview findings showed that some students in the experimental group were able to describe their perceived learning gains, emotion regulation methods, stress coping strategies, and possible applications of the course content. For example, students mentioned strategies or concepts such as “counting to 10,” observing facial cues, the concepts of the “upstairs brain” and “downstairs brain,” temporarily leaving the situation, discussing problems with others, and seeking support from family members.

## 4 Discussion

### 4.1 Research purpose

This study investigated the effects of integrating EEGs into emotion education curricula on fifth-grade students' learning achievement, emotion regulation strategies, and achievement emotions. Furthermore, it sought to clarify how GBL contexts may provide situated emotional practice to support the internalization and transfer of SEL strategies.

### 4.2 Major results

#### 4.2.1 The effects of educational escape games on learning achievement

This study found that EEG-integrated instruction significantly improved students' learning achievement in emotion education. The ANCOVA results showed that the learning gains of the experimental group were significantly higher than those of the control group, suggesting that EEGs may help improve students' performance on curriculum-based learning assessments. The classroom observation results further contextualized this quantitative finding. Compared with teacher-guided question-and-answer and explanation activities, students in the experimental group demonstrated a higher level of task concentration during the puzzle-solving tasks and more frequently engaged in task-related interactions, such as peer discussion, hint evaluation, answer negotiation, and active help-seeking. These observations suggest that EEGs may provide more opportunities for active engagement with course content through situated tasks and collaborative puzzle-solving activities, rather than merely requiring students to passively receive teacher explanations.

These findings align with prior GBL research (Alotaibi, 2024; Bodnar et al., 2016; Boyle et al., 2016; Gordillo and López-Fernández, 2024; Gyedu et al., 2026; Hainey et al., 2016; Hussein et al., 2019; López-Pernas et al., 2023; Plass et al., 2020; Tokac et al., 2019), supporting the view that GBL can promote learning performance through task contexts, interactive participation, and clear goals. Although the overall findings are consistent with previous studies, the results of this study also indicate that higher learning achievement does not necessarily coincide with higher enjoyment. In contrast to perspectives that emphasize positive emotions as a primary driver of learning (Pekrun et al., 2011), the experimental group significantly outperformed the control group in learning achievement despite decreased enjoyment and increased anxiety.

According to CVT (Pekrun, 2006), increased task difficulty and uncertainty may reduce perceived control and thereby elicit anxiety. However, when learning tasks also involve high challenge, clear goals, immediate feedback, and situational value, students may still invest effort and maintain task engagement. Furthermore, the challenge–threat biopsychosocial framework (van Gog et al., 2024) suggests that how individuals evaluate task demands in relation to their own resources influences whether a difficult task is interpreted as a challenge rather than a threat. As a result, even in the presence of negative emotional arousal, students may still engage in substantial mental effort.

Therefore, the findings of this study can be understood as follows: the challenge and time pressure embedded in EEGs may have increased students' anxiety, but these emotional responses did not necessarily lead to withdrawal. Classroom observations showed that, even when students in the experimental group experienced tension and a sense of urgency, they continued to discuss, check hints, and attempt to solve the puzzles. This suggests that, in a gamified context characterized by collaborative support, clear task goals, and room for error-tolerant practice, moderate task-related pressure may coexist with sustained engagement. In other words, this study does not argue that anxiety itself promotes learning. Rather, it suggests that EEGs may provide a distinctive learning context in which students can still achieve better curriculum-based learning performance through collaborative puzzle solving and sustained participation, even when they experience higher task-related anxiety.

#### 4.2.2 Dynamic shifts in emotion regulation strategies and intervention consistency

The second main finding concerns the effects of EEGs on changes in fifth-grade students' self-reported emotion regulation strategies during the emotion education course. The quantitative data showed that neither group demonstrated significant pretest–posttest changes in cognitive reappraisal or expressive suppression. After controlling for pretest scores, no significant between-group differences were observed. These results indicate that, during the 6-week intervention, this study did not observe significant short-term effects of the two instructional approaches on students' self-reported emotion regulation strategies. However, the internal consistency of the expressive suppression subscale was relatively low (Cronbach's α = 0.635), which may have reduced the ability of this study to detect changes in this dimension. Therefore, the nonsignificant findings related to expressive suppression should be interpreted with caution and should not be taken to imply that EEGs or traditional instruction had no effect on students' expressive suppression.

According to the process model of emotion regulation (Gross and John, 2003), although emotion regulation strategies may be activated or practiced in specific situations, changes in habitual strategy use generally require longer periods of time, repeated practice, and more stable contextual support (Gross, 2002; Gross and Thompson, 2007). Therefore, the absence of significant mean-level changes in this study may reflect the difficulty of immediately changing students' self-reported habitual emotion regulation tendencies through a short-term course intervention.

Although the quantitative results did not show significant changes, the focus group interviews provided supplementary insights into the subjective experiences of students in the experimental group. Some interviewed students mentioned that they were able to describe emotion regulation methods learned in the course, such as “counting to 10,” temporarily leaving the situation, shifting their thoughts, expressing emotions, or discussing problems with others. These responses suggest that some students in the experimental group were able to notice and describe possible emotion regulation methods and perceived the course content as helpful for understanding and regulating emotions. However, because the interview data were obtained from only seven students in the experimental group, these findings were used primarily to illustrate how the interviewed students understood the course content and emotion regulation strategies and how they described their learning experiences. They should therefore be interpreted with caution.

From the perspective of situated learning, EEGs may provide more contextualized opportunities for strategy practice than traditional explanation-based instruction (Brown et al., 1989). In this study, students in the experimental group were required to read hints, discuss answers, manage uncertainty, and attempt to solve problems within a context involving time limits, task challenges, and group collaboration. This activity structure may have allowed emotion education content to be presented not only as abstract concepts but also as ideas that students could encounter and consider within task-related pressure and peer interaction. In contrast, students in the control group learned the same course content primarily through teacher-guided question-and-answer and explanation activities, which may have provided fewer opportunities for contextualized practice and peer negotiation. This is also consistent with previous discussions of traditional lecture-based instruction, suggesting that when contextualized practice is limited, students may find it more difficult to transform abstract concepts into operational strategy understanding (Najjarpour, 2025).

In addition, the safe-failure mechanism in GBL may help reduce the psychological cost of trial and error (Loderer et al., 2020; Plass et al., 2015). In EEGs, students can attempt to solve puzzles, use hints, revise answers, and engage in further discussion in a context that does not produce real negative consequences. This error-tolerant learning environment may encourage students to continue participating in tasks even when they encounter frustration or uncertainty. Overall, this study did not observe that the short-term intervention immediately changed students' self-reported emotion regulation strategies. However, the qualitative data suggest that EEGs may provide students with contextualized experiences of emotional awareness and strategy understanding, thereby offering preliminary preparation for longer-term emotion regulation practice.

#### 4.2.3 Dynamic changes of achievement emotions

The third main finding concerns the effects of EEGs on changes in fifth-grade elementary school students' self-reported achievement emotions during the emotion education course. The quantitative data showed that, compared with the pretest, students in the experimental group reported higher anxiety and lower enjoyment after the course. After controlling for pretest scores, the experimental group also showed higher adjusted anxiety and lower adjusted enjoyment than the control group. These results suggest that the EEG context may have been accompanied by higher emotional arousal and lower enjoyment. However, because this study used a shortened version of the achievement emotions scale and the internal consistency of the anxiety subscale was relatively low (Cronbach's α = 0.571), the anxiety-related findings should be regarded as preliminary and exploratory self-reported emotional responses and interpreted with caution.

The qualitative data further provided contextual meaning for understanding the increase in students' self-reported anxiety. The focus group interviews showed that some students in the experimental group described anxiety or uneasiness mainly in relation to time pressure, puzzle difficulty, uncertainty about answers, and success or failure in completing the challenge. Classroom observations also showed that students in the experimental group often displayed tension and a sense of urgency when facing the countdown timer, difficult puzzles, or concerns that their group might not successfully complete the task. However, these emotional responses were generally accompanied by continued task engagement, such as continuing to discuss answers, checking hints, reminding group members to manage time, or attempting to solve the puzzles again, rather than withdrawal or giving up. Therefore, the anxiety observed in this study may be better understood as task-related pressure within the game-task context and should be interpreted in relation to students' sustained engagement and group collaboration.

This interpretation can be understood through control-value theory and the challenge–threat biopsychosocial framework. According to CVT (Pekrun, 2006), when task difficulty and uncertainty increase, students may experience anxiety due to reduced perceived control. However, when a task also involves clear goals, immediate feedback, peer support, and situational value, students may still maintain task engagement. The challenge–threat biopsychosocial framework also suggests that the way learners evaluate task demands relative to their available resources influences whether they interpret a stressful situation as a challenge or a threat (van Gog et al., 2024). In this study, the time pressure and puzzle-solving challenges embedded in EEGs may have increased students' anxiety, but collaborative puzzle solving, the hint mechanism, and task feedback may also have provided contextual resources that supported continued engagement. Therefore, this study does not argue that anxiety itself promotes learning. Rather, it suggests that, in a gamified context with supportive structures, task-related anxiety may coexist with sustained engagement.

The decrease in enjoyment requires more cautious interpretation. The experimental group reported a significant decrease in enjoyment after the course, indicating that EEGs did not produce a uniformly positive emotional experience for all students. This finding reminds us that GBL does not necessarily increase enjoyment. When tasks involve time limits, a sense of competition, puzzle difficulty, or the possibility of failure, some students may experience frustration, pressure, or emotional load, which may in turn reduce enjoyment (Loderer et al., 2020; Plass et al., 2015). In other words, although EEGs can provide situated, interactive, and challenging learning experiences, these design elements may also lead some students to experience higher psychological load, especially when they perceive insufficient time, difficult questions, or unsuccessful challenge completion. A more conservative interpretation is that the decrease in the experimental group's average enjoyment may reflect that some students experienced greater pressure or frustration during the game challenges, and that students may differ in their acceptance of and emotional responses to such challenges.

This finding also echoes reminders in GBL research regarding challenge design: when challenge is well matched with students' abilities, feedback, and support, it may promote engagement; however, when students experience insufficient control, it may undermine enjoyment (Pekrun, 2006; Plass et al., 2015). In the qualitative interviews, some students still described the course as “interesting” or as helping them “learn new knowledge,” suggesting that students' overall evaluations of the activity may simultaneously involve interest, a sense of achievement, pressure, and frustration. However, these positive descriptions should not be used to offset the quantitative finding of decreased enjoyment. Rather, they should be understood as indicating that students' emotional experiences were multifaceted and varied across individuals.

Overall, the achievement emotion findings of this study suggest that EEGs may create a high-challenge, high-arousal emotional learning context. Within this context, students may simultaneously experience higher task-related anxiety, lower enjoyment, and sustained task engagement. These findings remind us that when integrating GBL into emotion education or social and emotional learning courses, researchers and teachers should not simply assume that games will naturally elicit enjoyment or positive emotions. Instead, they need to carefully design task difficulty, time pressure, hint mechanisms, and peer support to prevent challenge from turning into excessive negative pressure and to help students face task-related emotions in a safe and supportive context.

### 4.3 Limitations and directions for future research

This study has several limitations. First, the intervention lasted only 6 weeks, and learning achievement and emotion-related variables were measured only immediately after the intervention. Therefore, this study could not examine the long-term effects of EEGs on students' learning achievement, emotion regulation strategies, and achievement emotions. Emotion regulation strategies involve response tendencies that gradually develop in students' daily contexts, and changes in these strategies generally require longer periods of time, repeated practice, and sustained support. Accordingly, the absence of significant changes in cognitive reappraisal and expressive suppression in this study may be partly attributable to the short intervention duration and the lack of follow-up data. Future research could adopt longer-term longitudinal designs and include delayed posttests to examine whether EEGs can produce more stable effects on students' learning achievement and emotion regulation strategies.

Second, the sample size of this study was relatively small and was drawn from only two intact classes in the same elementary school. Thus, the statistical power, external validity, and generalizability of the findings were limited. Because this study adopted a quasi-experimental design, the experimental and control groups were drawn from two existing classes. Students' data may therefore have involved within-class dependency, and class-level differences may have been confounded with intervention effects. Although both groups were taught by the same teacher and used the same emotion education course content to reduce the influence of teacher and curriculum differences, the inclusion of only two class clusters prevented the use of multilevel modeling and limited the ability to distinguish class-level variation from instructional intervention effects. Future studies should include more classes and schools and adopt cluster-randomized assignment or multilevel research designs to improve statistical power and the inferential strength of the findings.

Third, this study did not include a formal intervention fidelity check. Although the researchers-maintained consistency across the two instructional conditions through the same course content, the same classroom teacher, and standardized procedures, this study did not use systematic fidelity rating scales, classroom video coding, or external observer evaluations to confirm whether both instructional conditions were implemented fully as originally designed. Therefore, the possible effects of instructional quality, teacher facilitation, or differences in classroom interaction on the findings cannot be completely ruled out. Future research should include intervention fidelity checks, such as classroom observation scales, instructional procedure checklists, video analysis, or independent raters, to more precisely evaluate the quality of intervention implementation.

Fourth, the measurement instruments used in this study also had limitations. The learning achievement test was developed by the research team based on the 6-week emotion education course content and was reviewed by two elementary school teachers to confirm the appropriateness of the items for the course content and fifth-grade students' comprehension level. However, this study did not conduct formal item analysis or report reliability evidence for the learning achievement test. Therefore, the learning achievement results should be understood as curriculum-based learning performance rather than as results from a standardized achievement test. In addition, the same items were used for the pretest and posttest, which may have produced test familiarity effects. Although both the experimental and control groups completed the same pretest and posttest, and such testing effects may therefore have influenced both groups, the potential effect of repeated responding on posttest performance cannot be completely excluded. Future research could develop parallel test versions and conduct analyses of item difficulty, discrimination, and reliability to improve the rigor of learning achievement measurement.

Fifth, this study used self-report questionnaires and focus group interviews, which may have been affected by social desirability bias, students' verbal expression abilities, and recall bias. In addition, to reduce the response burden for elementary school students, this study used a shortened version of AEQ-ES, which also limited the psychometric strength of the measure. The enjoyment subscale included only two items, the anxiety subscale showed relatively low internal consistency (Cronbach's α = 0.571), and boredom was originally measured with only a single item; therefore, boredom was not included in the subsequent main analyses or theoretical interpretation. Given these limitations, the achievement emotion findings in this study were regarded as preliminary and exploratory self-reported emotional responses and were contextualized with qualitative data, rather than being used as the sole basis for strong causal or mechanistic conclusions. Future research could use the full version of the AEQ-ES or a validated short-form scale to improve the reliability and validity of measuring enjoyment, anxiety, and other achievement emotions. For the anxiety subscale, future studies could also expand the number of items to six to eight and re-examine its internal consistency, item structure, and criterion-related validity.

Sixth, the qualitative data in this study were mainly derived from focus group interviews with seven students in the experimental group. Because the sample scope was limited, these findings were primarily used to provide supplementary understanding of the subjective learning experiences of students in the experimental group and should not be generalized to all students or used as a basis for between-group comparison. Although classroom observations covered both the experimental and control groups, the observation data mainly reflected students' observable behaviors and interactions in the classroom and could not directly infer students' internal emotion regulation processes or their application of strategies outside the classroom. Future research could include interviews with more students, interviews with students in the control group, individual interviews, or learning process data to more fully understand students' emotional experiences and strategy use under different instructional conditions.

Finally, implementing EEGs requires teachers to invest time in activity design, game facilitation, and post-activity reflective discussion. Although standardized procedures help maintain curriculum consistency, the quality and timing of reflective discussion may still influence whether students can transform game experiences into emotion education learning content. Future instructional designs could include structured reflection modules, standardized questioning frameworks, and teacher training to reduce implementation burden and improve instructional quality. Future research could also further compare different game design conditions, such as the degree of collaboration, time pressure, hint mechanisms, and intensity of reflective discussion, to identify which design elements best support students' emotional awareness, task engagement, and emotion regulation learning. Despite these limitations, this study provides preliminary empirical evidence for the application of educational escape games in elementary school emotion education and social and emotional learning.

### 4.4 Conclusion

This study adopted a quasi-experimental mixed-methods design to examine the effects of integrating EEGs into an emotion education course on fifth-grade elementary school students' learning achievement, self-reported emotion regulation strategies, and self-reported achievement emotions. The results showed that, after controlling for pretest scores, students who participated in the EEGs performed significantly better on the curriculum-based learning achievement test than students who received teacher-guided question-and-answer activities and explanations. This finding suggests that EEGs may provide students with more opportunities to actively engage with emotion education content through situated tasks, collaborative puzzle solving, clear goals, and immediate feedback.

However, this study did not observe significant short-term changes in students' self-reported emotion regulation strategies. This finding reminds us that changes in emotion regulation strategies may require longer periods of time, repeated practice, and sustained support, and that a 6-week intervention may be insufficient to immediately change students' habitual self-reported tendencies toward cognitive reappraisal and expressive suppression. The qualitative data showed that some students in the experimental group were able to describe emotion regulation methods learned in the course and perceived these contents as helpful for understanding and regulating emotions. Therefore, EEGs may be more appropriately understood as a contextualized practice environment for emotional awareness and strategy understanding, rather than as a single short-term intervention capable of producing stable changes in emotion regulation tendencies.

Regarding achievement emotions, students in the experimental group reported higher anxiety and lower enjoyment. Because this study used a shortened version of AEQ-ES and the internal consistency of the anxiety subscale was relatively low, these findings should be regarded as preliminary and exploratory self-reported emotional responses and interpreted with caution. The qualitative data further showed that students' anxiety during the game was mainly related to time pressure, puzzle difficulty, uncertainty about answers, and success or failure in completing the challenge. These emotional responses were usually accompanied by continued participation, group discussion, and attempts to solve the puzzles, rather than withdrawal or giving up. In other words, this study does not argue that anxiety itself promotes learning. Rather, it suggests that, in a gamified context with collaborative support, hint mechanisms, and room for error-tolerant practice, task-related anxiety may coexist with sustained engagement.

Overall, this study provides preliminary empirical evidence for the application of EEGs in elementary school emotion education and social and emotional learning. The findings suggest that well-designed EEGs may help improve students' learning performance in emotion education and provide contextualized experiences through which students can notice and understand emotions during task challenges. At the same time, this study reminds us that GBL does not necessarily lead to higher enjoyment. If task difficulty, time pressure, or support mechanisms are not appropriately designed, students may experience greater pressure and lower enjoyment. Therefore, when integrating EEGs into emotion education courses, teachers and researchers should pay attention to the balance among task challenge, hint mechanisms, peer collaboration, and reflective discussion in order to create a gamified learning environment that supports both learning effectiveness and emotional support.

## Data Availability

The raw data supporting the conclusions of this article will be made available by the authors, without undue reservation.
