# Supplementary material for: Educational escape games in emotion education: effects on learning achievement, emotion regulation strategies, and achievement emotions among upper elementary students
Source: Front Psychol. 2026 Jul 14;17:1877695. doi: 10.3389/fpsyg.2026.1877695 (PMC13408268; doi:10.3389/fpsyg.2026.1877695)
Supplement: Supplementary file 1 [file Supplementary_file_1.docx]

***Supplementary Material 1***

The SEL intervention curriculum consisted of six weekly sessions. Table 1 summarizes the curriculum structure, instructional sequence, learning objectives, core content, and activity design for each week. The curriculum was organized in a progressive manner, beginning with basic emotional awareness and the relationship between emotions and the brain, followed by emotional and bodily responses, empathy and active listening, the connection among thoughts, emotions, and behaviors, anger regulation, and stress coping strategies. This structure was intended to help students develop emotional awareness, interpersonal understanding, and practical emotion regulation and coping skills throughout the intervention.

**Table 1** Structure, sequence, and instructional content of the six-week SEL intervention curriculum

| **Week** | **Course Topic** | **Course Focus /Learning Objectives** | **Main Content** | **Teaching Methods / Activity Design** |
| --- | --- | --- | --- | --- |
| Week 1 | Understanding Emotions and the Brain | This session established the foundational concepts of emotional education. Students learned what emotions are and understood the relationship between rational thinking and emotional responses through the concepts of the “upstairs brain” and the “downstairs brain.” | Establishing classroom expectations; introducing the goals of the emotional education course; understanding the definition of emotions; explaining the concepts of the “upstairs brain” and the “downstairs brain”; learning to identify emotions and emotion-related behavioral responses. | Teacher explanation, video viewing and discussion, examples from daily life situations, use of a hand model to explain the brain, and story-based discussion. |
| Week 2 | Emotional Experiences and Bodily Responses | This session helped students further develop awareness of the connection between emotions and bodily sensations, and learn to use an “emotion thermometer” to evaluate emotional intensity. | Understanding that emotions involve not only psychological experiences but also bodily changes; distinguishing between comfortable and uncomfortable feelings; understanding emotional intensity; learning to evaluate the strength of emotions. | Video-guided instruction, group discussion using emotion picture cards, ball-passing game, student sharing, situational question discussion, and emotion thermometer activity. |
| Week 3 | Empathy and Active Listening | The focus of this session shifted to interpersonal interaction. Students learned to empathize with others and understand others’ emotions through active listening. | Understanding the importance of empathy; learning to observe emotional cues such as facial expressions, tone of voice, and body language; learning the “six aspects” of active listening: eyes, ears, heart, hands, feet, and mouth. | Story-based guidance, identification of facial expressions from pictures, group responses, situational performance, role-playing, and teacher–student demonstration of active listening. |
| Week 4 | Thoughts, Emotions, and Behaviors | This session emphasized the relationship between cognition and emotions, helping students understand that different thoughts may lead to different emotions and behaviors. | Recognizing that thoughts influence emotions; understanding that the same event may lead to different emotions and behaviors depending on one’s thoughts; learning to respect oneself and others; understanding that thoughts and emotions can be changed after calming down. | Viewing a video on “Sai Weng Lost His Horse,” question discussion, situational performance, table-based analysis of emotions, thoughts, and respect, group responses, and teacher-led synthesis. |
| Week 5 | Anger Patterns and Emotion Regulation | This session focused on the emotion of anger. Students learned about the process of anger and practiced concrete and actionable emotion regulation strategies. | Understanding the anger pattern of “triggering event–emotion–behavior–consequence”; recognizing maladaptive ways of handling anger; learning emotion regulation strategies, such as noticing emotions, understanding causes, counting to ten, leaving the situation, and self-talk. | Teacher explanation, situational performance, group discussion, guided question-and-answer activities, case analysis, and practice of emotion regulation strategies. |
| Week 6 | Stress and Coping Strategies | This session extended the course to the issue of stress. Students learned to distinguish between healthy and unhealthy stress and practiced positive coping strategies. | Understanding stress, healthy stress, unhealthy stress, stressors, realistic and unrealistic expectations, and stress responses; learning healthy and unhealthy coping strategies for dealing with stress. | PPT-based situational picture prompts, explanation of key terms, discussion of stressful situations, strategy classification table, situational question practice, and course summary. |
