# Supplementary material for: Educational escape games in emotion education: effects on learning achievement, emotion regulation strategies, and achievement emotions among upper elementary students
Source: Front Psychol. 2026 Jul 14;17:1877695. doi: 10.3389/fpsyg.2026.1877695 (PMC13408268; doi:10.3389/fpsyg.2026.1877695)
Supplement: Supplementary file 2 [file Supplementary_file_2.docx]

***Supplementary Material 2***

Table 1 presents the weekly puzzle design used in the six-week educational escape game intervention. Each week included six puzzle items aligned with the corresponding SEL instructional topic and learning objectives. The puzzles were designed with a gradual increase in difficulty, progressing from basic concept recall to situational application and integrative understanding. For each puzzle item, the table provides the key focus, puzzle question and response options, correct answer, difficulty level, source of difficulty, and hints that could be provided to students when they encountered challenges. This design was intended to support students’ engagement with the core SEL concepts taught each week while providing structured scaffolding through hints during the escape game activities.

**Table 1** Weekly puzzle design, difficulty progression, and student hints for the educational escape game intervention

| ***Week 1: Understanding Emotions and the Brain*** | | | | | | |
| --- | --- | --- | --- | --- | --- | --- |
| **Item** | **Key Focus** | **Question and Options** | **Answer** | **Difficulty** | **Source of Difficulty** | **Hint for Students** |
| 1 | Definition of emotion | What is an emotion?  A. Only anger is considered an emotion.  B. A psychological feeling generated by internal or external stimuli.  C. Emotions only occur when someone cries.  D. Emotions only appear during exams. | B | Easy | Students only need to recall the basic concept of emotions. | Think about this: When you feel happy, angry, scared, or sad, what kind of feeling appears in your mind? |
| 2 | Function of the upstairs brain | In the course, what was the “upstairs brain” mainly responsible for?  A. Producing emotions.  B. Helping us stay calm, think, and make plans.  C. Helping us sleep.  D. Making us feel hungry. | B | Easy | This item mainly involves recall of a course-specific term. | Think about this: Is the “upstairs brain” more like a helper for calm thinking or a helper for immediate impulsive reactions? |
| 3 | Function of the downstairs brain | In the course, what was the “downstairs brain” mainly responsible for?  A. Emotional reactions.  B. Writing essays.  C. Solving mathematics problems.  D. Memorizing texts. | A | Easy | This item mainly involves recall of a course-specific term. | Think about this: When people feel very scared or angry and react before they have time to think, which part may be more active? |
| 4 | Emotion and rational thinking | If someone breaks your eraser and you feel angry, but you choose to calmly tell the person how you feel, what are you using?  A. Rational thinking of the upstairs brain.  B. An explosive reaction of the downstairs brain.  C. Having no emotion at all.  D. A method of ignoring others. | A | Moderately difficult | Students need to apply the concept of the “upstairs brain” to a new daily-life situation. | Think about this: If you feel angry but can still speak calmly, does that mean you first used calm thinking? |
| 5 | Consequence of the story | In the story, a junior high school student felt very happy when he saw his classmate and threw a small stone at the window. What happened as a result?  A. The classmate was happy, and they played together.  B. The window broke, and compensation was required.  C. The teacher praised him for being creative.  D. Everyone forgot about the incident. | B | Moderate | Students need to recall the content of the story and the result of the event. | Recall the story: After the junior high school student threw the small stone, what happened to the window? How did the adults handle it? |
| 6 | Lesson of the story | What is the main lesson of the story?  A. Having emotions is bad.  B. When we are happy, we can do anything we want.  C. Emotions are natural, but emotion-related behavioral responses should be appropriate.  D. We should always shout when we are angry. | C | Difficult | Students need to infer the core concept behind the story rather than simply recalling the story content: emotions are natural, but behavioral responses should be appropriate. | Think about this: The story is not saying that we should not have emotions. Instead, it reminds us that after emotions appear, what should we choose appropriately? |
| ***Week 2: Emotional Experiences and Bodily Responses*** | | | | | | |
| **Item** | **Key Focus** | **Question and Options** | **Answer** | **Difficulty** | **Source of Difficulty** | **Hint for Students** |
| 1 | Relationship between emotions and bodily sensations | What is the relationship between emotions and bodily sensations?  A. They are completely unrelated.  B. Emotions only affect the face and do not affect the body.  C. Emotions are often accompanied by bodily responses.  D. Only happiness involves bodily sensations. | C | Easy | Students only need to understand that emotions are accompanied by bodily responses. | Think about this: When you feel nervous, do you experience bodily sensations such as a faster heartbeat or sweaty palms? |
| 2 | Identifying bodily responses | Which of the following may be a bodily response when someone feels nervous?  A. A faster heartbeat.  B. No feeling at all.  C. Hair suddenly growing longer.  D. Eyes turning blue. | A | Easy | The options are clearly different, making the answer easy to identify. | First eliminate impossible options, such as hair suddenly growing longer or eyes changing color. |
| 3 | Purpose of the roller coaster video | Why was the roller coaster video used in class?  A. To show that roller coasters are fun.  B. To help students understand that emotions and bodily sensations may occur together.  C. To show that everyone must like roller coasters.  D. To show that people never feel scared on roller coasters. | B | Moderate | Students need to understand that the video itself was not the focus; rather, it was used to guide students to understand emotions and bodily responses. | The teacher did not show the video only to let everyone watch an amusement ride. The purpose was to help students observe possible bodily responses when people feel scared or excited. |
| 4 | Emotional intensity | What does “emotional intensity” mean?  A. Emotions can sometimes be strong and sometimes weak.  B. Everyone’s emotions are equally strong.  C. Only anger has intensity.  D. Emotional intensity cannot change. | A | Moderate | Students need to understand that emotions are not simply present or absent; they can also vary in strength. | Think about this: Is it possible to feel “a little angry” or “very angry”? |
| 5 | Emotion thermometer | Xiaoming is going to the zoo tomorrow and feels very happy. What tool can he use to judge the strength of his emotion?  A. Emotion thermometer.  B. Ruler.  C. Scale.  D. Calculator. | A | Moderately difficult | Students need to apply the “emotion thermometer” to a new situation. | Recall the tool used in class to measure the strength of emotions. Its name includes the word “thermometer |
| 6 | Individual differences | According to the course, how might people’s emotional intensity differ when facing the same event?  A. It must be exactly the same.  B. It may be very different.  C. No one will have emotions.  D. Only teachers will feel differently. | B | Difficult | Students need to understand individual differences in emotions and recognize that not everyone feels the same way in response to the same event. | Think about this: Some people like roller coasters, while others are scared of them. Will everyone feel exactly the same about the same event? |
| ***Week 3: Empathy and Active Listening*** | | | | | | |
| **Item** | **Key Focus** | **Question and Options** | **Answer** | **Difficulty** | **Source of Difficulty** | **Hint for Students** |
| 1 | Story situation | Xiaohua lost his hair because of medical treatment, and his classmates laughed at him at first. What did this behavior mainly lack?  A. Empathy.  B. Memory.  C. Physical strength.  D. Imagination. | A | Easy | Students can judge from the story situation that the behavior lacked empathy. | Think about this: When someone loses hair because of illness, is laughing at him a way to understand his feelings or a failure to take his perspective? |
| 2 | Supportive behavior | Later, all the classmates shaved their heads together. What was the main purpose?  A. To make the teacher angry.  B. To support Xiaohua and help him feel that he was not alone.  C. Because the weather was too hot.  D. Because everyone did not want to attend class. | B | Easy | Students can directly understand the supportive behavior in the story. | Recall the story: Did everyone shave their heads to help Xiaohua feel accompanied, or to laugh at him? |
| 3 | Definition of empathy | Which of the following is closest to the meaning of “empathy”?  A. Only thinking about one’s own feelings.  B. Truly feeling, understanding, and supporting another person’s emotions.  C. Asking everyone to listen only to oneself.  D. Ignoring what happens to others. | B | Moderate | Students need to understand that empathy is not simply sympathy, but involves understanding and supporting another person’s feelings. | Empathy is not just saying, “You are so pitiful.” It means trying to understand another person’s feelings. |
| 4 | Emotional cues | Which of the following can help us notice another person’s emotions?  A. Facial expressions.  B. Tone of voice.  C. Body language.  D. All of the above. | D | Moderate | Students need to integrate different emotional cues, including facial expressions, tone of voice, and body language. | When observing another person’s emotions, you can look at the face, listen to the tone of voice, and pay attention to body movements. |
| 5 | Six aspects of active listening | Which of the following is not included in the “six aspects” of active listening?  A. Eyes.  B. Ears.  C. Heart.  D. Sleeping. | D | Moderately difficult | Students need to remember the six aspects of active listening and exclude the incorrect option. | Recall that the “six aspects” are all ways to help us listen to others. Which option is unrelated to listening? |
| 6 | Application of active listening | A friend says, “I was supposed to go to the movies, but my mother asked me to stay home and do practice tests.” Which response best reflects active listening?  A. “You are really unlucky,” and then turning to talk to someone else.  B. “Let me show you my toy.”  C. “You sound angry and sad because you were looking forward to the movie but cannot go.”  D. “Stop talking. I’m busy.” | C | Difficult | Students need to identify a response that reflects the friend’s emotion and the reason for that emotion, rather than choosing ordinary comfort. | A good listening response usually mentions how the other person may feel and why the person may feel that way. |
| ***Week 4: Thoughts, Emotions, and Behaviors*** | | | | | | |
| **Item** | **Key Focus** | **Question and Options** | **Answer** | **Difficulty** | **Source of Difficulty** | **Hint for Students** |
| 1 | Main idea of the story | What was the main purpose of the story “Sai Weng Lost His Horse”?  A. To show that horses are important.  B. To show that emotions are related to thoughts.  C. To show that all villagers are smart.  D. To show that exercise is important. | B | Easy | Students only need to grasp the connection between the story and the course theme. | Think about this: In the story of “Sai Weng Lost His Horse,” the same event might later turn out to be good or bad. This is related to how we think about things. |
| 2 | Differences in thoughts | When the same event happens, different people may have different emotions mainly because of differences in what?  A. Thoughts.  B. Height.  C. Pencils.  D. Seats. | A | Easy | The concept is direct and the answer is clear. | The same event may make one person feel disappointed and another person feel fine. What is mainly different between them? |
| 3 | Function of calming down | When something unpleasant happens, what can calming down first help us do?  A. Understand our own thoughts more clearly.  B. Scold others immediately.  C. Forget the event completely.  D. Make the problem worse. | A | Moderate | Students need to understand that calming down can help them rethink the situation. | Think about this: People tend to act impulsively when they are very angry. After calming down, what can they do better? |
| 4 | Judging respect | During a class trip discussion, a student says, “Going to the zoo is childish! How old are you?” This response is:  A. Respectful of others’ opinions.  B. Disrespectful of others’ opinions.  C. Very empathetic.  D. The best way to communicate. | B | Moderate | Students need to judge whether the statement shows respect for others. | Listen to the sentence: “Going to the zoo is childish!” Does this sentence make people feel comfortable? Does it respect others’ opinions? |
| 5 | Appropriate response | Which of the following behaviors better respects both oneself and others?  A. Yelling loudly at a classmate.  B. Breaking someone else’s things immediately when angry.  C. Taking a short break or counting from 1 to 10 when feeling irritated.  D. Drawing on the wall with crayons when feeling anxious. | C | Moderately difficult | Students need to choose an appropriate strategy from several emotional responses. | First eliminate options that hurt oneself, hurt others, or damage things. |
| 6 | Integration of relationships | What does this lesson teach us that thoughts can influence?  A. Emotions and behaviors.  B. Shoe size.  C. Physical education class time.  D. Classroom lighting. | A | Difficult | Students need to integrate the relationship among thoughts, emotions, and behaviors. | Recall the key point of this lesson: When thoughts about the same event differ, the following emotions and behaviors may also differ. |
| ***Week 5: Anger Patterns and Emotion Regulation*** | | | | | | |
| **Item** | **Key Focus** | **Question and Options** | **Answer** | **Difficulty** | **Source of Difficulty** | **Hint for Students** |
| 1 | Sequence of the anger pattern | What is the usual sequence of the anger pattern?  A. Triggering event → emotion → behavior → consequence.  B. Consequence → behavior → emotion → triggering event.  C. Behavior → triggering event → consequence → emotion.  D. Emotion → sleeping → eating → attending class. | A | Easy | This item involves recall of a process taught in class. | Recall that the anger pattern begins with “something happening,” followed by an emotion, then a behavior, and finally a result. |
| 2 | Identifying anger | Xiaotang accidentally tore a book borrowed from Aqiang. Later, he heard that all the classmates knew about it. What might he feel?  A. Very angry.  B. Nothing at all.  C. Very sleepy.  D. Looking forward to class. | A | Easy | Students can judge the emotion based on the situation. | Think about this: If something you did not want others to know became known to the whole class, how might you feel? |
| 3 | Consequences of behavior | If Xiaotang immediately scolds Xiaomi and ends their friendship because he is angry, what consequence might occur?  A. Losing a friend.  B. The problem will definitely be solved immediately.  C. Everyone will become happier.  D. The book will repair itself. | A | Moderate | Students need to understand that angry behaviors can affect interpersonal relationships. | Think about this: If you scold a friend and end the friendship because you are angry, will the friendship become better or worse? |
| 4 | Maladaptive anger management | Which of the following is a maladaptive way to handle anger?  A. Counting to 10.  B. Taking a walk.  C. Hitting others or shouting.  D. Saying to oneself, “Relax.” | C | Moderate | Students need to distinguish between healthy and unhealthy ways of handling emotions. | First identify which option may hurt others, scare others, or make the problem worse. |
| 5 | Emotional awareness | When we notice that we are very angry, which of the following is more appropriate?  A. Slamming the door immediately.  B. First noticing and becoming aware of our own emotion.  C. Laughing at the other person immediately.  D. Avoiding the problem forever. | B | Moderately difficult | Students need to understand that the first step in emotion regulation is to notice emotions rather than react immediately. | The first step in emotion regulation is to know whether you are feeling angry, sad, or nervous. |
| 6 | Purpose of the strategy | What is the main purpose of “counting from 1 to 10”?  A. To help oneself pause and have time to think of a better way.  B. To make oneself angrier.  C. To scare others.  D. To show that one is good at mathematics. | A | Difficult | Students need to understand the function behind the strategy: pausing, calming down, and gaining time to think, rather than simply remembering the strategy name. | Counting to 10 is not for practicing mathematics. It helps you pause for a moment and avoid reacting impulsively right away. |
| ***Week 6: Stress and Coping Strategies*** | | | | | | |
| **Item** | **Key Focus** | **Question and Options** | **Answer** | **Difficulty** | **Source of Difficulty** | **Hint for Students** |
| 1 | Definition of stress | What is stress?  A. A bodily sensation or emotional tension.  B. Something only adults experience.  C. Something that only happens during sleep.  D. Something that does not affect us at all. | A | Easy | This item involves recall of a basic concept. | Think about this: Before an exam, competition, or presentation, what do we call the tense feeling that may appear in the mind or body? |
| 2 | Healthy stress | Xiaoyan reviewed his lessons over the weekend. Although he felt pressure during a quiz, he was confident. What type of stress is this more likely to be?  A. Healthy stress.  B. Unhealthy stress.  C. No stress at all.  D. Stress that cannot be handled. | A | Moderate | Students need to judge whether the stress in the situation helps performance. | Xiaoyan prepared in advance. Although he felt pressure, could the pressure help him face the task more seriously? |
| 3 | Unhealthy stress | Xiongxiong played video games all weekend and did not review. During the quiz, he could hardly answer the questions and broke out in a cold sweat. What type of stress is this more likely to be?  A. Healthy stress.  B. Unhealthy stress.  C. No stress.  D. Happy stress. | B | Moderate | Students need to judge that the stress has caused negative reactions and distress. | Xiongxiong did not prepare, and the stress made him break out in a cold sweat and almost unable to answer. Is this stress helping him or troubling him? |
| 4 | Healthy coping strategy | Which of the following is a healthy coping strategy for stress?  A. Discussing difficulties with friends or family members.  B. Drinking alcohol or using drugs.  C. Spending a long time online to avoid homework.  D. Getting angry at family members or friends. | A | Moderate | Students need to distinguish between healthy and unhealthy coping strategies. | Healthy ways of handling stress usually do not hurt oneself or others. |
| 5 | Application to a stressful situation | You will participate in a speech contest in three days and feel very stressed. Which of the following is a healthier response?  A. Practicing at a fixed time every day.  B. Giving up.  C. Keep playing video games and procrastinating.  D. Overeating. | A | Moderately difficult | Students need to apply stress-coping strategies learned in class to a new situation. | The speech contest is coming soon. The most helpful approach is usually to practice and prepare, rather than avoid the task. |
| 6 | Integration of stress regulation | When we feel stressed, what does the course remind us to do?  A. Find positive solutions and allow ourselves to relax appropriately.  B. Do nothing about it.  C. Yell at others.  D. Escape in unhealthy ways. | A | Difficult | Students need to integrate key course concepts: finding positive solutions and relaxing appropriately rather than avoiding or attacking. | When you feel stressed, you can find ways to solve the problem and also relax appropriately. First eliminate options involving avoidance, aggression, or self-harm. |
