# Supplementary material for: Educational escape games in emotion education: effects on learning achievement, emotion regulation strategies, and achievement emotions among upper elementary students
Source: Front Psychol. 2026 Jul 14;17:1877695. doi: 10.3389/fpsyg.2026.1877695 (PMC13408268; doi:10.3389/fpsyg.2026.1877695)
Supplement: Supplementary file 3 [file Supplementary_file_3.docx]

***Supplementary Material 3***

**Learning Effectiveness Pretest and Posttest**

Instructions: This section is mainly to understand your "Emotional Intelligence". There are 20 questions in each test. Please choose the answer that best fits your understanding. There is only one correct answer for each question.

**Table 1** Pretest: Emotional Intelligence

| No. | Question Item | Options |
| --- | --- | --- |
| 1 | What do we call the psychological and physiological feelings generated by internal or external stimuli? | (1) Perception (2) Emotion (3) Disturbance (4) Affect |
| 2 | Tian-Tian cried because she did poorly on a quiz. Which emotion is she most likely feeling? | (1) Joy (2) Calm (3) Anger (4) Sadness |
| 3 | Pang-Hu often gets furious over small matters. Which aspect of his life is LEAST likely to be affected? | (1) Singing ability (2) Physical health (3) Interpersonal relationships (4) Mental health |
| 4 | When facing difficulties, Feng-Feng thinks: "This is a test for me, proving how resilient I am." What emotion is she LEAST likely to feel? | (1) Disturbance (2) Shame (3) Boredom (4) Calm |
| 5 | Xiao-Yu was angry with his mother and threw his belongings around. Which part of his brain failed to exert control? | (1) Upstairs Brain (2) Downstairs Brain (3) Whole body (controllable) (4) Whole body (uncontrollable) |
| 6 | What is the main theme of this curriculum? | (1) Venting emotions (2) Emotional awareness (3) Emotion regulation (4) Recognizing emotions |
| 7 | Regarding the concept of “emotions,” which of the following is INCORRECT? | (1) There is no right or wrong regarding emotions (2) Multiple emotions can occur at once (3) There are many types of emotions (4) Behavior from emotions has no right/wrong |
| 8 | Active listening includes the “Six Arrivals” (sensory/mental focus). Which is NOT one of them? | (1) Ears (2) Heart (3) Nose (4) Feet |
| 9 | Xuan-Xuan thought after an argument: “This is a great chance for us to communicate.” Which emotion is she LEAST likely to feel? | (1) Boredom (2) Calm (3) Happiness (4) Peace |
| 10 | Why is respecting oneself and others important? | (1) Teacher said so (2) Disrespect ruins relationships (3) Treat others as you want to be treated (4) Disrespect leads to poor grades |
| 11 | What is empathy? | (1) Genuinely feeling others’ emotions and willing to help (2) Treating everyone with the same attitude (3) Judging events from the person’s perspective and deciding for them (4) A mindset of helping the vulnerable |
| 12 | Which is a good “emotion regulation” method? | (1) Mocking a sibling when they get punished (2) Staying silent when misunderstood by a teacher (3) Observing one’s own emotions when a friend is busy (4) Yelling at a sibling for breaking a sticker |
| 13 | Which reaction helps improve a situation? | (1) Counting 1 to 10 to calm down (2) Refusing to speak to a rude classmate (3) Scribbling on a desk when stressed (4) Making a sibling trip because they made you trip |
| 14 | Shan-Shan hoped her parents would stop arguing. What emotion is she MOST likely feeling? | (1) Sadness (2) Happiness (3) Calm (4) Pride |
| 15 | What is “emotion regulation”? | (1) Ability to handle anger, stress, or anxiety (2) Ability to recognize emotions (3) Ability to handle positive emotions (4) Following one’s impulses |
| 16 | Why is learning “emotion regulation” important? | (1) To transform many emotions into one (2) To understand oneself (3) It is related to our future quality of life (4) To avoid danger |
| 17 | “Show-off Grandpa” likes to flaunt his things. What is the likely reason? | (1) He enjoys showing off (2) Lack of self-confidence (3) High capability (4) Fear of parental reprimand |
| 18 | An uncle handled a business crisis calmly despite his anxiety. Which part of his brain was functioning? | (1) Upstairs Brain (2) Downstairs Brain (3) Emotions (4) Body |
| 19 | If you meet a “Gossip Demon,” what feeling are they LEAST likely to bring to others? | (1) Boredom (2) Happiness (3) Fear (4) Anger |
| 20 | Which belongs to the “Leaving the Scene” method of emotion regulation? | (1) Reporting a clerk’s poor attitude to the manager (2) Staying silent and going to one’s room after being scolded (3) Reflecting on the causes of feeling annoyed |

**Table 2** Posttest: Emotional Intelligence

| No. | Question Item | Options |
| --- | --- | --- |
| 1 | What do we call feelings generated by stimuli? | (1) Emotion (2) Perception (3) Friendship (4) Disturbance |
| 2 | Da-Lun trembled when seeing photos of reptiles. Which emotion is he likely feeling? | (1) Peace (2) Anger (3) Fear (4) Sadness |
| 3 | A-Mo was late to class to avoid heavy schoolwork and had a stomachache. What is LEAST likely to be affected? | (1) Physical health (2) Interpersonal relationships (3) Drama/Acting ability (4) Mental health |
| 4 | A sister thinks of a setback as: “An opportunity to improve my resilience.” What is she MOST likely feeling? | (1) Happiness (2) Boredom (3) Sadness (4) Anger |
| 5 | Xiao-Ze was angry at his 4-year-old brother and threw his toys. Which part of his brain failed to control the bad decision? | (1) Upstairs Brain (2) Downstairs Brain (3) Whole body (controllable) (4) Whole body (uncontrollable) |
| 6 | What is the main theme of this curriculum? | (1) Recognizing emotions (2) Emotion regulation (3) Venting emotions (4) Emotional awareness |
| 7 | Regarding “emotions,” which is INCORRECT? | (1) There are many types of emotions (2) Multiple emotions can occur at once (3) Behavior from emotions has no right/wrong (4) Emotions themselves have no right/wrong |
| 8 | What is empathy? | (1) Treating everyone with the same attitude (2) A mindset of helping the vulnerable (3) Judging events from the person’s perspective and deciding for them (4) Genuinely feeling others’ emotions and willing to help |
| 9 | A-Jun was one point away from a perfect score and thought: “It’s a pity I was careless.” What is he LEAST likely to feel? | (1) Happiness (2) Boredom (3) Regret (4) Sadness |
| 10 | What is a “Show-off Grandpa” LEAST likely to bring to people around him? | (1) Fear (2) Sadness (3) Happiness (4) Anger |
| 11 | Active listening includes the “Six Arrivals.” Which is NOT one of them? | (1) Mouth (2) Ears (3) Eyes (4) Nose |
| 12 | Why is respecting oneself and others important? | (1) School rules (2) Fear of parents (3) To earn others’ respect (4) To avoid teacher punishment |
| 13 | Which reaction helps improve a situation? | (1) Dismissing class because of heat (2) Counting 1 to 10 to calm down when misunderstood (3) Hitting a wall when anxious (4) Deducting points from a teammate because they cost you points |
| 14 | Xiao-Sheng thought: “This communication might improve my parents’ relationship.” What is he MOST likely feeling? | (1) Boredom (2) Sadness (3) Fear (4) Peace |
| 15 | What is “emotion regulation”? | (1) Ability to handle stress, anger, or anxiety (2) Ability to handle positive emotions (3) Ability to understand emotions (4) Following one’s impulses |
| 16 | Why is learning “emotion regulation” important? | (1) Teacher said so (2) To recognize others’ emotions (3) To understand oneself (4) It is related to our future quality of life |
| 17 | Why does a “Gossip Demon” mock others’ weaknesses? | (1) They naturally enjoy it (2) They have no strengths (3) They think others will be happy (4) They lack self-confidence |
| 18 | An aunt handled a farm crisis calmly despite her anxiety. Which part of her brain was functioning? | (1) Upstairs Brain (2) Downstairs Brain (3) Farm (4) Body |
| 19 | Which is a good “emotion regulation” method? | (1) Hitting a friend for ruining a notebook (2) Staying silent when a father takes money (3) Ruining a sister’s bag because she ruined yours (4) Observing and feeling one’s own emotions after a poor test |
| 20 | Which belongs to the “Leaving the Scene” method of emotion regulation? | (1) Going to one’s room after being scolded (2) Asking a father for a room break when feeling low (3) Reflecting on the causes of feeling annoyed (4) Scolding a sister for taking toys |
