# Supplementary material for: Educational escape games in emotion education: effects on learning achievement, emotion regulation strategies, and achievement emotions among upper elementary students
Source: Front Psychol. 2026 Jul 14;17:1877695. doi: 10.3389/fpsyg.2026.1877695 (PMC13408268; doi:10.3389/fpsyg.2026.1877695)
Supplement: Supplementary file 4 [file Supplementary_file_4.docx]

***Supplementary Material 4***

**Emotion Regulation Questionnaire**

Note: Participants indicated their level of agreement with each statement using a 5-point Likert scale.

**Table 1** Pretest and Posttest Items: Emotion Regulation

| Dimension | No. | Item Description | Strongly Agree 5 | Agree 4 | Neutral 3 | Disagree 2 | Strongly Disagree 1 |
| --- | --- | --- | --- | --- | --- | --- | --- |
| Cognitive Reappraisal | 1 | I control my emotions by changing the way I think about the situation I am in. | □ | □ | □ | □ | □ |
| Expressive Suppression | 2 | I keep my feelings to myself. | □ | □ | □ | □ | □ |
| Cognitive Reappraisal | 3 | When I want to feel happier, I think about something different. | □ | □ | □ | □ | □ |
| Expressive Suppression | 4 | When I am positive, I am careful not to show it too much. | □ | □ | □ | □ | □ |
| Cognitive Reappraisal | 5 | When I am feeling bad, I try to think about something that makes me feel better. | □ | □ | □ | □ | □ |
| Expressive Suppression | 6 | I control my emotions by not expressing them. | □ | □ | □ | □ | □ |
| Cognitive Reappraisal | 7 | I think about things in a way that helps me stay calm when I am upset. | □ | □ | □ | □ | □ |
| Cognitive Reappraisal | 8 | I try to understand the reasons behind my emotions to help me manage them. | □ | □ | □ | □ | □ |
| Expressive Suppression | 9 | When I am feeling bad, I make sure I do not show it. | □ | □ | □ | □ | □ |
| Cognitive Reappraisal | 10 | When I am happy, I think about why I am happy to maintain the feeling. | □ | □ | □ | □ | □ |
