# Supplementary material for: Educational escape games in emotion education: effects on learning achievement, emotion regulation strategies, and achievement emotions among upper elementary students
Source: Front Psychol. 2026 Jul 14;17:1877695. doi: 10.3389/fpsyg.2026.1877695 (PMC13408268; doi:10.3389/fpsyg.2026.1877695)
Supplement: Supplementary file 5 [file Supplementary_file_5.docx]

***Supplementary Material 5***

**Achievement Emotions Questionnaire**

Note: This instrument assesses three dimensions of achievement emotions (Enjoyment, Anxiety, and Boredom) related to the emotion education curriculum. Participants indicated their level of agreement with each statement using a 5-point Likert scale. Items marked with (R) are reverse-coded.

**Table 1** Pretest Items: Achievement Emotions

| Dimension | No. | Item Description | Strongly Agree 5 | Agree 4 | Neutral 3 | Disagree 2 | Strongly Disagree 1 |
| --- | --- | --- | --- | --- | --- | --- | --- |
| Enjoyment | 1 | I look forward to learning about emotion education. | □ | □ | □ | □ | □ |
|  | 2 | I would rather postpone this boring activity until tomorrow. (R) | □ | □ | □ | □ | □ |
| Anxiety | 3 | I am so nervous that I do not even want to start learning. | □ | □ | □ | □ | □ |
|  | 4 | I start feeling uneasy when I have to study the materials. | □ | □ | □ | □ | □ |
|  | 5 | I feel anxious when I see how much content I still have to learn. | □ | □ | □ | □ | □ |
| Boredom | 6 | I do not want to study because I feel bored. | □ | □ | □ | □ | □ |

**Table 2** Posttest Items: Achievement Emotions

| Dimension | No. | Item Description | Strongly Agree 5 | Agree 4 | Neutral 3 | Disagree 2 | Strongly Disagree 1 |
| --- | --- | --- | --- | --- | --- | --- | --- |
| Enjoyment | 1 | I am so happy about the progress I have made that I am motivated to continue learning. | □ | □ | □ | □ | □ |
|  | 2 | Reflecting on my progress in the emotion education activities makes me feel very happy. | □ | □ | □ | □ | □ |
| Anxiety | 3 | I worry about whether I have correctly understood the content of these activities. | □ | □ | □ | □ | □ |
|  | 4 | It scares me when I find myself unable to keep up with the activities. | □ | □ | □ | □ | □ |
|  | 5 | I am concerned that what I have learned is insufficient to complete my learning plan. | □ | □ | □ | □ | □ |
| Boredom | 6 | Certain topics were so enjoyable that I was motivated to read additional related information. (R) | □ | □ | □ | □ | □ |
