# Supplementary material for: Educational escape games in emotion education: effects on learning achievement, emotion regulation strategies, and achievement emotions among upper elementary students
Source: Front Psychol. 2026 Jul 14;17:1877695. doi: 10.3389/fpsyg.2026.1877695 (PMC13408268; doi:10.3389/fpsyg.2026.1877695)
Supplement: Supplementary file 6 [file Supplementary_file_6.docx]

***Supplementary Material 6***

**Semi-structured Interview Guide**

I. Introduction

1. Explain the purpose of this study to the students and promise that all content will be kept confidential. The data presented in the thesis will be anonymized to ensure that students can share their thoughts comfortably and without concern.
2. Invite students to share their positive or negative thoughts or behaviors during this semester, emphasizing that no value judgments will be made regarding the content.
3. Invite students to share any situations or changes they noticed within the class this semester, and further inquire if they noticed any transformations in specific classmates.
4. Invite students to share their emotion regulation processes and methods in daily life, encouraging them to speak freely without worrying about whether their methods are "correct."

II. Interview Outline

1. In this emotion education curriculum, which part did you enjoy the most? Why? What specific methods or skills did you learn?
2. Do you use these methods in your daily life? How do you apply them?
3. How did you feel before participating in this emotion education curriculum? How do you feel now after completing the curriculum?
4. As a senior elementary student, you face significant academic pressure. When you encounter high-pressure situations, what emotions do you experience? How do you handle these emotions?
5. Following up on the previous question, do you express these emotions (through facial expressions, words, or body language)? What methods do you use to help yourself transform or shift these emotions?
6. After completing the emotion education curriculum, in what ways do you believe this course has been helpful to you?
